# Stem cell-derived extracellular vesicles in the therapeutic intervention of Alzheimer's Disease, Parkinson's Disease, and stroke

**DOI:** 10.7150/thno.95953

**Published:** 2024-05-27

**Authors:** Wantong Zhou, Xudong Wang, Yumeng Dong, Peifen Gao, Xian Zhao, Mengxia Wang, Xue Wu, Jiuheng Shen, Xin Zhang, Zhiguo Lu, Wenlin An

**Affiliations:** 1 National Vaccine Serum Institute (NVSI), China National Biotech Group (CNBG), Sinopharm Group, No. 38 Jing Hai Second Road, Beijing 101111, China.; 2 Capital Medical University, 10 Xitoutiao, Youanmenwai Street, Beijing 100069, China.; 3 State Key Laboratory of Biochemical Engineering, Institute of Process Engineering, Chinese Academy of Sciences, Beijing 100190, China.

**Keywords:** stem cell, extracellular vesicle, neurological disease, drug delivery, clinical translation

## Abstract

With the increase in the aging population, the occurrence of neurological disorders is rising. Recently, stem cell therapy has garnered attention due to its convenient sourcing, minimal invasiveness, and capacity for directed differentiation. However, there are some disadvantages, such as poor quality control, safety assessments, and ethical and logistical issues. Consequently, scientists have started to shift their attention from stem cells to extracellular vesicles due to their similar structures and properties. Beyond these parallels, extracellular vesicles can enhance biocompatibility, facilitate easy traversal of barriers, and minimize side effects. Furthermore, stem cell-derived extracellular vesicles can be engineered to load drugs and modify surfaces to enhance treatment outcomes. In this review, we summarize the functions of native stem cell-derived extracellular vesicles, subsequently review the strategies for the engineering of stem cell-derived extracellular vesicles and their applications in Alzheimer's disease, Parkinson's disease, and stroke, and discuss the challenges and solutions associated with the clinical translation of stem cell-derived extracellular vesicles.

## 1. Introduction

As the aging population has steadily expanded in recent decades, the incidence of Alzheimer's Disease (AD), Parkinson's Disease (PD), and stroke has continued to rise annually 1. These conditions impact either the central or peripheral nervous systems, leading to impairments in brain function, spinal cord function, neuromuscular function, and peripheral nerves. These diseases are characterized by neuroinflammation, oxidative stress, mitochondrial dysfunction, impaired neuronal cells, disrupted blood-brain barrier (BBB) function, and the accumulation of abnormal proteins, resulting in lasting neurological deficits 2. Despite remarkable strides and breakthroughs in pharmacological treatments, surgical techniques, and rehabilitation modalities over the last fifty years, there is still no permanent solution; treatments only offer relief from symptoms and delay disease progression 3.

Stem cells (SCs), a category of undifferentiated cells with the potential for diverse specialization, self-replication, and self-renewal, represent a promising strategy. Their advantageous features, including a convenient source, minimal invasiveness, portability, and the ability to undergo directed differentiation in various inductive environments, underscore their potential in the realm of neurological therapeutics 4. Various studies over the past few decades have shown that stem cell transplantation can promote neurogenesis and facilitate nerve healing post-injury 5. However, stem cell application encounters significant limitations due to the quality control, immune incompatibility, safety evaluation, ethical considerations and logical considerations, and tumorigenicity 6.

Extracellular vesicles (EVs) are small bilayer lipid structures discharged by most eukaryotic cells and tissue types 7-9. Possessing structures and properties akin to cells, EVs present a distinctive advantage. SC-EVs differ from stem cells in that they neither replicate nor undergo uncontrolled division, which helps avoid issues related to the use of stem cells, such as the risk of tumor formation and the challenges of successful engraftment 10. Importantly, SC-EVs possess the ability to cross the BBB to generate therapeutic impacts within the brain 11. In addition, SC-EVs enhance the longevity and availability of therapeutic cargo in EV-based nanocarriers compared to EVs derived from other cells, thanks to the immuno-regulating characteristics acquired from the parent cells 12. It has been also indicated that SC-EVs contribute to favorable outcomes, including extending therapeutic effects, stimulating the immune system, enhancing quality control, and maintaining long-term storage at -80°C 13. Furthermore, biomedical engineering technology can additionally optimize both the exterior and interior of SC-EVs, enabling them to target particular cells, enhance efficient BBB crossing, and attain specific therapeutic results 14, 15. Inspiringly, the treatment efficacy of SC-EVs has been extensively explored in diverse neurological disorder models.

In this review, we summarize the methods of obtaining SC-EVs, including the isolation and differentiation of stem cells, and isolation and purification of extracellular vesicles. Then, we review the functions of native SC-EVs, including neuroprotection, angiogenesis and preservation BBB integrity, alleviation of neuroinflammation, and other functions. However, there are drawbacks including poor targeting efficiency, inconsistent therapeutic outcomes, and limited output efficiency, which can be solved by precondition, loading drug, and modified surface. Consequently, we summarize the strategies for the engineering of SC-EVs and their applications in AD, PD, and stroke. Ultimately, we outline the challenges linked to these extracellular vesicles in clinical translation and offer potential solutions.

## 2. From stem cell therapy to stem cell-derived extracellular vesicle therapy

The identification of SCs in the second half of the 19th century, recognizing them as an inherent mechanism for the body's development and self-repair, marked a revolutionary turning point in medical practice. This pivotal discovery substantially heightened interest in researching stem cells for the treatment of AD, PD, and stroke 16. SCs are undifferentiated cells distinguished by remarkable attributes such as elevated proliferation, differentiation, and self-renewal capacities, setting them apart from specialized cells 17. At present, SCs are commonly derived from four primary origins. The primary origins encompass (1) embryonic tissue, (2) fetal tissues (including the umbilical cord, amniotic fluid, and placenta), (3) particular sites within the mature organism, such as adipose, blood, bone marrow, skin, or skeletal muscle, and (4) differentiated somatic cells following genetic reprogramming, specifically induced pluripotent stem cells (iPSCs) 18. SCs have various therapeutic potentials in addressing central nervous system (CNS) disorders, and they can contribute to neural injury repair through several mechanisms. Firstly, SCs can migrate to damaged neural sites, replacing dead or damaged nerve cells through a cell replacement mechanism, thereby repairing the compromised neural network. Then, SCs have the capability to secrete a significant quantity of active and nutrient factors, thereby stimulating neural cells, facilitating the regeneration and formation of new cells, and releasing angiogenic factors to encourage vascular generation at the site of injury. Moreover, SCs can modulate the immune response by adjusting the number of immune cells reaching the pathological site and secreting different levels of cytokines that mutually influence each other, providing a protective anti-inflammatory effect 19. Nevertheless, the application of SCs faces notable limitations attributed to challenges in quality control, high cost, potential complications, one-off effects, as well as storage and transportation 20.

**Figure 1:**
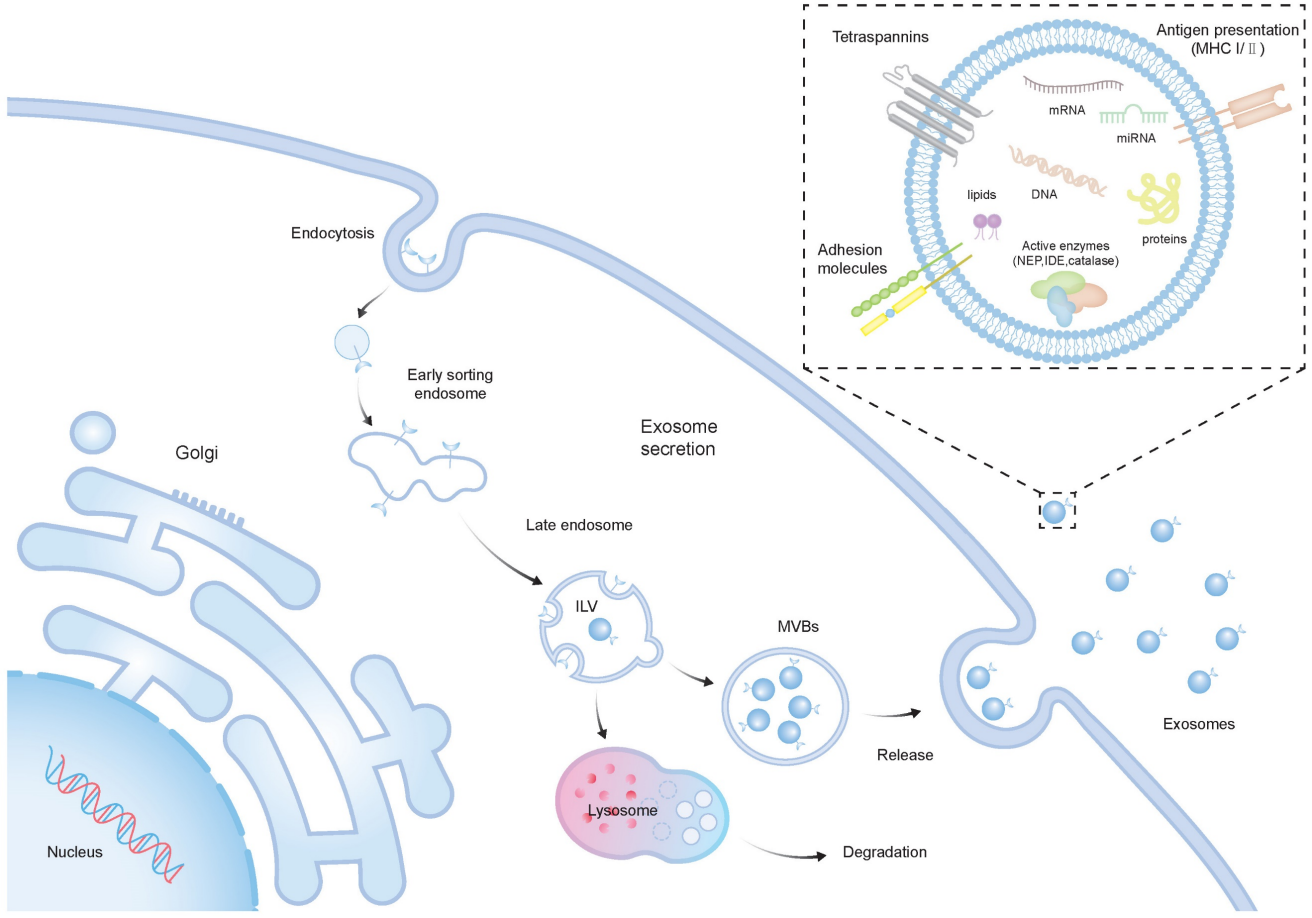
** The biogenesis and structure of exosomes.** The biogenesis of exosomes initiates with the inward budding of the plasma membrane, resulting in the creation of early sorting endosomes (ESEs), which may subsequently mature into LEs and MVBs. MVBs can either fuse with lysosomes for degradation or convey ILVs to the plasma membrane, releasing them into the extracellular space, thus forming exosomes. Exosomes are lipid membrane-enclosed extracellular structures, which include a variety of cellular proteins, lipids, DNAs, and RNAs with diverse biological functions.

Recent investigations suggest that in models of neurological conditions, the treatment impact of SCs is associated with the paracrine mechanism, particularly involving EVs 21. EVs, lipid membrane-enclosed extracellular structures, are typically released by various cell types. Moreover, they are frequently found in a range of bodily fluids, including breast milk, blood, and urine 22. EVs encapsulate a rich cargo inherited from their parent cells, comprising cytosolic proteins, lipids, DNAs, and RNAs 23, 24. They consist of exosomes (30-100 nm in diameter), microvesicles (200-1000 nm), and apoptotic bodies (500-5000 nm) 25. The intricate process of EV biogenesis, with a primary focus on exosomes, mainly involves the internalization of the membrane to form early endosomes, and the maturation of early endosomes into late endosomes, resulting in the development of intraluminal vesicles (ILVs) within large multivesicular bodies (MVBs) 7. The majority of ILVs are expelled into the extracellular environment through a process where they merge with the cell's plasma membrane, resulting in the formation and release of exosomes 26 (Figure 1). As of now, over 9700 proteins, 3400 mRNAs, and 2800 miRNAs have been recognized within EVs. There is a diverse array of proteins that serve various functions in EVs from endosomes, peripheral membrane, and the cytosol, including receptor tyrosine kinases, and cytosolic signaling proteins 27. The surface of EVs is adorned with numerous membrane proteins, among them annexins, flotillins, and tetraspanins, including CD81, CD9, CD63 28.

In the context of regenerative medicine, SC-EVs have several advantages compared to cell-based therapies. An important benefit lies in the potential for SC-EVs to exhibit lower immunogenicity compared to their parent cells, contingent upon their origin. This could be attributed to the diminished presence of transmembrane proteins like MHC complexes on their surfaces 29. In contrast to traditional nanocarriers such as liposomes, SC-EVs demonstrate superior biocompatibility and reduced immunogenicity. These properties enable them to evade recognition and clearance by the host immune system following administration 30, 31. Additionally, SC-EVs do not undergo replication following injection, reducing the risk of neoplastic growth and the latent transmission of viral pathogens 10. Notably, SC-EVs demonstrate inherent capabilities to traverse tissue and cellular barriers, enabling their penetration through the BBB 11. Furthermore, SC-EVs may exhibit intrinsic targeting properties, demonstrating an affinity for specific cells or tissues. This characteristic can be utilized to precisely transport drugs to their intended targets, thus minimizing off-target effects 32. Moreover, in contrast to live cells, SC-EVs boast an extended shelf life, facilitating prolonged transport and storage capabilities. Interestingly, the surface and contents of SC-EVs can be finely tuned through advanced engineering and editing technologies to improve the therapeutic effects 33. Numerous further instances highlight the successful preclinical applications of SC-EVs, with some summarized in subsequent sections of this review.

## 3. Methods of obtaining SC-EVs

### 3.1 Isolation and differentiation of SCs

Recent technical advances enable the acquisition of SCs through three distinct methods: (1) direct isolation from primary tissues, such as fetal tissues and specific sites within the adult organism; (2) differentiation from pluripotent stem cells, including induced pluripotent stem cells (iPSCs) and embryonic stem cells (ESCs); (3) trans-differentiation from somatic cells, which could be subsequently genetically reprogrammed 18.

Isolation of SCs from various tissues can be carried out using a range of methods that rely on varied physical, chemical, or surface molecule-based attributes of cells. These methods may involve considerations such as cell size and density, the presence of distinct cell surface markers, or cell adhesion to synthetic substrates.

Principal physical separation techniques, including gradient centrifugation method, cell adherence method and membrane filtration method, are influenced by the physical properties of cells such as size, density and adherence. These methods are rapid, simple and cost-effective. Nevertheless, these techniques are primarily utilized for enriching SCs from the tissue or for initial separation, which is then followed by more precise separation techniques such as fluorescence-activated cell sorting (FACS) and magnet-activated cell sorting (MACS) 34, 35. Immunoaffinity separation techniques are highly specific methods that leverage the unique expression of surface molecules on cells. These techniques utilize monoclonal antibodies that are tailored to bind with precision to these molecules. The antibodies are conjugated to either fluorescent markers or magnetic particles, enabling the targeted cells to be selectively identified and separated. While these methods offer high precision and specificity, they also face challenges related to cost-effectiveness and meeting specific market demands 36.

Besides, SCs can be differentiated from ESCs and iPSCs. For example, the application of synthetic coatings has demonstrated efficacy in facilitating the derivation of induced mesenchymal stem cells (iMSCs). Employing a synthetic polymer coating, poly 2-(methacryloyloxy) ethyl dimethyl-(3-sulfopropyl) ammonium hydroxide, during the derivation process from iPSCs resulted in a notable high differentiation efficiency 37. Likewise, Chen *et al.* illustrated that the treatment of iPSCs with SB431542, an inhibitor of the transforming growth factor beta pathway, in a two-dimensional (2D) culture system led to the generation of epithelial monolayer-like cells. Subsequent induction of epithelial-mesenchymal transition under these conditions resulted in rapid and reliable differentiation into iMSCs 38. Regarding neural differentiation, pluripotent SCs can be broadly categorized into two primary pathways: embryoid body (EB) formation and monolayer culture 39. Morizane *et al.* demonstrated that inhibitors can downregulate bone morphogenetic protein and TGFβ/activin/nodal signaling pathways, thereby promoting neural lineage specification 40. Surmac *et al.* also indicated that the Notch-related transmembrane protein Dlk1 can facilitate the neural progenitor differentiation through Notch signaling 41.

Lastly, SCs can be trans-differentiated from somatic cells. Song *et al.* proposed a comprehensive protocol for generating neural-like progenitors from MSCs derived from bone marrow and umbilical cord blood 42.

### 3.2 Isolation and purification of SC-EVs

The isolation of SC-EVs holds paramount importance in unraveling their biological activity mechanisms and unlocking their potential applications in treatment. Various isolation methods are selected for different purposes and applications, including ultracentrifugation, size-based isolation techniques, polymer precipitation, and immunoaffinity capture techniques, which are among the commonly employed options 43. Methods for isolating and purifying SC-EVs from culture medium or biological fluids are summarized in Table 1.

At present, ultracentrifugation (UC) is widely regarded as the isolation technique and is considered the gold standard for extracting and separating SC-EVs, including differential and gradient ultracentrifugation. UC primarily isolates the necessary components by leveraging the disparities in size and density among the various constituents in the starting mixture, rendering it well-suited for separating components with substantial differences in sedimentation coefficient 51. Differential ultracentrifugation effectively isolates SC-EVs and can yield high-purity samples. Nonetheless, the process of subjecting samples to repeated rounds of centrifugation, especially when high centrifugal forces are used, may result in damage to the vesicles that is not readily reversible 52. As for gradient ultracentrifugation, SC-EVs migrate to specific density layers according to their size and density, allowing for further purification and separation of EV subpopulations. However, the high viscosity of the sucrose solution reduces the rate of sedimentation for SC-EVs, leading to longer processing times 53.

A novel method for isolating SC-EVs from large samples involves utilizing size-exclusion chromatography (SEC). The separation mechanism behind SEC is based on the principle that larger macromolecules are unable to penetrate the gel pores and are instead eluted alongside the mobile phase through the gaps between the porous gels, while smaller molecules are trapped within the gel pores until they are eluted by the mobile phase 44. SEC offers a rapid, straightforward, and cost-effective means of isolating SC-EVs. This method ensures that the SC-EVs retain their intact structure, consistent size, and experience minimal changes to their inherent biological properties. However, there is a possibility of contamination with particles of comparable size, leading to a potential decrease in purity 43.

Ultrafiltration serves as an alternative size-based separation technique that operates on the principle of membrane filtration, where SC-EVs are separated based on their size through membranes with different pore diameters. Ultrafiltration is simpler and faster than ultracentrifugation, does not require specialized equipment, and can readily be scaled up for clinical applications involving extracellular vesicles. However, ultrafiltration may sometimes result in EV damage due to shear forces applied. This can be minimized by carefully adjusting the pressure applied to the membrane 54.

Moreover, the method of polymer precipitation has been reported to produce SC-EVs comparable to the gold standard of the differential centrifugation. The method of polymer precipitation commonly utilizes polyethylene glycol (PEG) to facilitate the collection of SC-EVs. This process involves the reduction of the SC-EVs' solubility, followed by their isolation through centrifugation. The polymer precipitation method offers ease of operation with a quick analysis time and is well-suited for handling large sample volumes. Nevertheless, it is associated with relatively lower purity and recovery rates, potential false positives, and the challenge of removing the polymer residue, which may hinder subsequent functional experimental analysis 55.

Furthermore, immunoaffinity chromatography, a method for separation and purification, relies on the precise interaction between antibodies and ligands to extract desired substances from heterogeneous mixtures. This technique ensures robust specificity, remarkable sensitivity, exceptional purity, and a high yield. However, its practical application is hindered by the high cost and the availability of specific antibodies 56.

In general, while every approach has its advantages and disadvantages, their drawbacks can be mitigated by integrating multiple purification techniques to improve both the quality and quantity.

## 4. The functions of native SC-EVs

Various studies have illustrated that administering SC-EVs can lead to the favorable outcomes in animals, including neuroprotection, angiogenesis and preservation of BBB integrity, alleviation of neuroinflammation, and other functions. The focus is particularly on MSC-EVs and NSC-EVs (Table 2).

### 4.1 Neuroprotection

Neuroprotection refers to strategies and interventions aimed at preserving the stability of the CNS microenvironment, as well as preserving and safeguarding the health and function of the nervous system, particularly the brain. According to the research, alterations in neurotrophic factor expression or their receptors are believed to contribute to neuronal deterioration and are involved in the pathogenesis of neurological diseases 86. Inspiringly, EVs offer a promising therapeutic avenue for neurological disorders, as they are capable of transporting essential neurotrophic factors to the brain and stimulating neuroprotective mechanisms 87. These neurotrophic factors (NTFs) are a class of endogenous biomolecules that include brain-derived neurotrophic factor (BDNF), nerve growth factor (NGF), and glial cell-derived neurotrophic factor (GDNF). They are pivotal in driving cell proliferation and differentiation within the nervous system, contributing to the maintenance and repair of neural tissues 88. Moreover, molecular analysis has convincingly demonstrated the presence of NTFs in EVs derived from MSCs 89, 90. It has been demonstrated that EVs derived from MSC therapy work as paracrine effectors responsible for promoting functional recovery through proteomics analysis of the EVs that identified many proteins that could be implicated in brain repair function 91. For example, Liu *et al.* exhibited that administration of BMSC-EVs via the lateral ventricle enhanced AD-like behavioral performance in mice injected with streptozotocin (STZ). The enhancements are likely due to the influence on glial cell activation, which leads to a decrease in neuroinflammatory responses, as well as the positive impact of BDNF related neuropathological changes within the hippocampal region 58. Several studies have shown that NSC-EVs exhibit a therapeutic effect similar to MSC-EVs by transporting neurotrophic factors, including BDNF, NGF, and GDNF 92.

**Figure 2:**
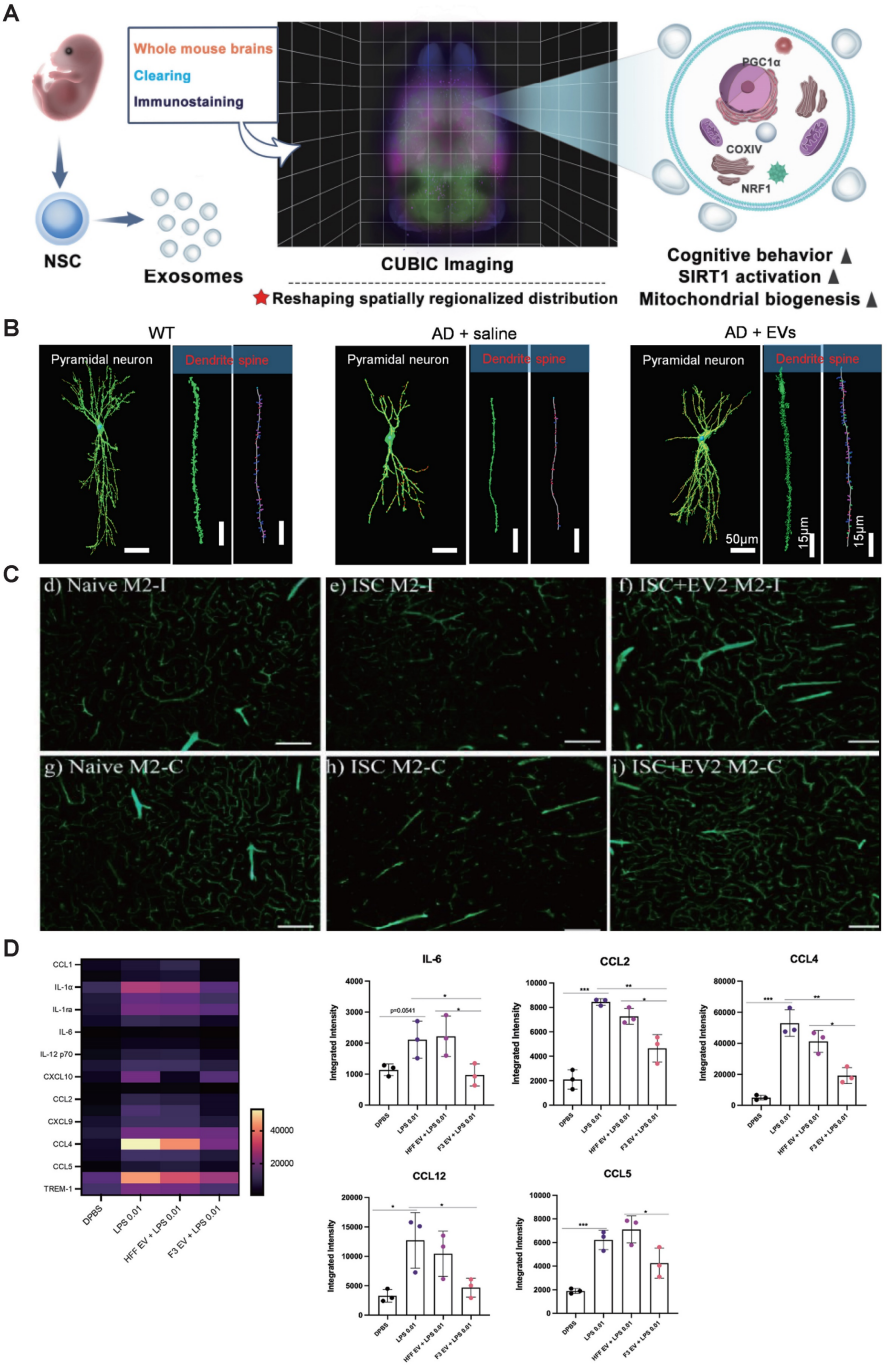
** The functions of native SC-EVs.** (A) NSC-EVs remodel the abnormal distribution of mitochondrial biogenesis-related proteins throughout the brain and enhance mitochondrial function. Adapted with permission from 62, copyright 2023 Medknow. (B) The administration of MSC-EVs restores hippocampal neuronal morphology in mice. Adapted with permission from 57, copyright 2021 Springer Nature. (C) The parameters of brain blood vessels were evaluated in the region of the supplementary motor cortex following the administration of extracellular vesicles on the 42nd day post-treatment. Adapted with permission from 74, copyright 2021 MDPI. (D) Anti-inflammatory effect of EVs on LPS-treated cells. Copyright 2022 Lee E. J. *et al.* Adapted with permission from 66, copyright 2022 BMC.

Some researches revealed that EVs administered are effectively assimilated by dopaminergic neurons within the affected hemisphere of the brain. EVs demonstrated significant efficacy in ameliorating damage to the nigral-striatal dopamine system while concurrently mitigating microglial activation 64. Wang *et al.* revealed that MSC-EVs effectively ameliorated cognitive impairments and neuronal loss. The involvement of the Nrf2 signaling pathway was observed in the mechanisms underlying the actions of MSC-EVs in APP/PS1 mice (Figure 2B) 57. Besides, He *et al.* conducted a study assessing the impact of T-MSC-EVs, which are enriched with miR-100-5p, on dopamine neuron damage and oxidative stress in both MPTP-induced PD mouse models and MPTP-induced MN9D cells. The T-MSC-EVs were found to directly target the 3' untranslated region of the NOX4 gene. This specific targeting mechanism was shown to provide neuroprotection against the degeneration of dopamine neurons, maintain the integrity and functionality of the nigrostriatal pathway, reduce motor impairments, and decrease oxidative stress via the Nox4-ROS-Nrf2 signaling pathway in PD models 63. Accumulating evidences have shown that treatment with MSC-derived EVs markedly ameliorated in vivo ischemia/reperfusion brain injury, enhanced neuron viability in vitro, and reduced apoptosis. Liu *et al.* demonstrated that MSC-EVs attenuated neuronal injury caused by oxygen and glucose deprivation (OGD) through the IL-33/ST2 signaling pathway in astrocytes, thereby reducing ischemia-induced brain injury in a mouse model 78. Interestingly, MSC-EVs exhibited upregulation of miR-22-3p, and inhibition of EV-miR-22-3p led to heightened apoptosis and reduced neuronal survival. Mechanistically, miR-22-3p derived from MSC-EVs mitigated ischemic brain injury by suppressing KDM6B-mediated actions on the BMP2/BMF axis 67. Exosomes enriched with KLF4 have shown considerable promise in mitigating the impact of MCAO in mouse models, effectively decreasing infarct size, neuronal injury, and apoptosis. This therapeutic effect is realized through the influence on the lncRNA-ZFAS1/FTO/Drp1 signaling axis. Importantly, KLF4 originating from BMSCs promotes the elevation of long non-coding RNA ZFAS1 levels. This increase in ZFAS1 expression subsequently leads to the inhibition of Drp1's m6A methylation, a regulatory event carried out by the RNA demethylase FTO 68. Besides, MSC-EVs facilitated the restoration of functional neurological capacity and brain tissue restructuring in aged rats following a stroke. They effectively mitigated motor-coordination deficits in both young and aged rats at both doses administered. Notably, in aged rats, EVs significantly decreased brain macrophage infiltration. Additionally, EV treatment promoted neurogenesis in the subventricular zone 69.

### 4.2 Angiogenesis and preservation BBB integrity

SC-EVs additionally contribute to the therapeutic effect through improving angiogenesis. Angiogenesis, the process of forming new blood vessels, serves as a vital physiological defense mechanism critical for replenishing oxygen and nutrient supply to injured brain tissue after ischemic events. By promoting blood vessel growth, angiogenesis holds promise in stabilizing brain perfusion and boosting neuronal survival, neurological recovery, and brain plasticity 93. Xue *et al.* demonstrated that MSC-derived EVs maintain the transcriptional activity of human brain microvascular endothelial cells (HBMECs), which represent a predominant component of the brain's microvasculature. Additionally, MSC-derived EVs were observed to facilitate the recovery in a PD mouse model by promoting angiogenesis related to ICAM1 65. Hu *et al.* found that BMSC-EVs significantly improved neurological function, decreased the size of cerebral infarcts, and boosted the density of microvessels along with the expression of miR-21-5p in the context of cerebral ischemia. Additionally, in vitro studies revealed that BMSC-EVs also enhanced the functionality of human umbilical vein endothelial cells (HUVECs), as evidenced by increased proliferation, migration, and tube formation capabilities 70.

The BBB is a highly selective and protective barrier which controls the blood circulation to the brain and the CNS, preventing potentially harmful exogenous molecules from entering the brain and maintaining homeostasis. Thus, it assumes a pivotal role in both the initiation and management of AD, PD, and stroke 94. Interestingly, MSC-EVs effectively traversed the BBB and exhibited selective accumulation within injured brain regions. Subsequently, these accumulated MSC-EVs mitigated tPA-induced BBB disruption and reduced hemorrhage by suppressing astrocyte activation and inflammation in murine ischemic stroke models. Mechanistically, miR-125b-5p, delivered by MSC-EVs, played a pivotal role in preserving BBB integrity through its targeting of TLR4 and inhibition of NF-kB signaling in astrocytes 75. Some evidence showed that a single dose of EVs administered intranasally 24 hours post-focal permanent ischemic stroke in rats led to significant improvements. These improvements included preservation of BBB integrity, and enhanced vascular stability within the salvageable peri-infarct region. Moreover, there was a noteworthy decrease in the volume of the infarct (Figure 2C) 74. Li *et al.* demonstrated that administrations of both brain endothelial cell (BEC) and BMSC-derived EVs improved cerebral infarction, BBB integrity, and neurological deficits in MCAO rats. Furthermore, BMSC-EVs showed comparable inhibitory impacts to BEC-EVs on the Cav-1-mediated endocytosis of ZO-1 and Claudin-5. Notably, BMSC-EVs were found to suppress Cav-1 expression and improve neurological function 73. Additionally, the elevated levels of CD147, Caveolin-1, vascular endothelial growth factor A (VEGFA), and vascular endothelial growth factor receptor 2 (VEGFR2) in brain microvessels were decreased following EVs administration. Moreover, metalloproteinase (MMP) activity inhibition was observed. 71. Similarly, several studies have demonstrated that NSC-EVs successfully preserve BBB integrity in various disease models. For example, Zhang *et al.* discovered that EVs enhance poststroke BBB integrity by decreasing the expression of ATP-binding cassette transporter B1 (ABCB1) and downstream matrix MMP-9 activity in stroke mice 76.

### 4.3 Alleviation of neuroinflammation

Neuroinflammation is a key contributor to neurological diseases and injuries, and excessive inflammation can lead to neuronal damage. In patients suffering from neurological diseases, it is common to observe aberrant activation of astrocytes and microglia, along with elevated levels of pro-inflammatory molecules 95. Emerging evidence has proven that SC-EVs play an important role in anti-inflammatory. The accumulation of MSC-EVs in the hemisphere ipsilateral to the occlusion leads to a reduction in injury volume, providing protection during the sub-acute injury phase. By 72 h, MSC-sEVs in the IN group were primarily localized within Iba1+ cells with retracted processes, as well as within GLUT1+ blood vessels in regions subjected to ischemic-reperfusion 77. Treatment with MSC-EVs markedly decreased infarct volume, alleviated behavioral deficits, and mitigated microglial activation three days after transient brain ischemia. Zhang *et al.* demonstrated that miR-146a-5p contained within hUMSC-EVs diminishes microglia-mediated neuroinflammatory responses by targeting the IRAK1/TRAF6 pathway 81. Lee *et al.* found that the intracerebral injection of NSCs-EVs into the substantia nigra (SN) region of PD model mice led to a noteworthy reduction in neuroinflammation in that area. This reduction was associated with a significant decrease in the presence of activated microglia, reactive astrocytes, and pro-inflammatory factors within both the striatum and SN region, which is crucial for PD treatment (Figure 2D) 66.

The NLRP3 inflammasome, a complex assembly of proteins, is integral to the innate immune system's response to invading pathogens and signals of cellular distress by initiating the release of pro-inflammatory cytokines 96. Some studies have demonstrated that SC-EVs can mitigate neuroinflammation by inhibiting NLRP3. For example, the intracisternal administration of iPSC-MSC-EVs effectively mitigated neuroinflammation and ameliorated cognitive deficits in an AD mouse model. This beneficial effect is attributed to the inhibition of NLRP3/GSDMD-driven neuroinflammation by miR-223-3p 59. Additionally, NSC-EVs, enriched with Y box binding protein (YBX1), have been demonstrated to augment the stability of the m6A-modified G protein-coupled receptor 30 (GPR30) via interaction with IGF2BP1. This stabilization leads to an upregulation of GPR30, which in turn suppresses NLRP3 inflammasome activation by promoting the ubiquitination of NLRP3 by the speckle-type POZ protein (SPOP). Consequently, this mechanism significantly contributes to the suppression of neuronal pyroptosis in the context of ischemic stroke 83.

There is a wealth of research indicating that EVs alleviate the recruitment of inflammatory cells. EVs significantly reduced leukocyte infiltration, particularly polymorphonuclear neutrophils, monocytes, and macrophages, within the ischemic brain tissue of older mice. Additionally, MSC-EVs have caused a noteworthy reduction in the peripheral blood levels of monocytes and activated T lymphocytes 79. However, the immunomodulatory effects of EVs are subject to dynamic changes and have a finite duration. Zheng *et al.* indicated that NSC-EVs had no impact on leukocytes, monocytes, B cells, or T cells in MCAO mice. Instead, NSC-EVs were discovered to counteract post-ischemic peripheral immunosuppression, resulting in elevated levels of T and B lymphocytes in the bloodstream 69.

### 4.4 Other functions

Pathological proteins, exemplified by amyloid-β (Aβ) plaques and hyperphosphorylated tau, play a central role in AD pathology, which results in synaptic disruption and neuronal degeneration. The substantial benefits of removing these abnormal proteins are evident in the treatment of AD 97. Recent research has revealed that MSC-EVs delivered miR-29c-3p to neurons, effectively inhibiting β-site amyloid precursor protein cleaving enzyme 1 (BACE1) expression while activating the Wnt/β-catenin pathway. This dual mechanism led to increased expression of Aβ decomposition-related factors (NEP and IDE), reduced formation of amyloid-β (Aβ) plaques and deposition areas, as well as lowered levels of Aβ1-42 and inflammatory cytokine levels 60. Similarly, Cone *et al.* demonstrated that a reduced Aβ plaque burden was noted in the hippocampus of mice treated with EVs. Notably, diminished colocalization between glial fibrillary acidic protein (GFAP) and Aβ plaques was evident in the brains of EV-treated mice relative to those treated with saline 61.

SC-EVs also exert a therapeutic effect enhancing the differentiation of oligodendrocyte progenitor cells (OPCs). Fibrinogen deposition hindered remyelination post-MCAO by impeding oligodendrocyte progenitor cells (OPC) differentiation through the activation of ACVR1, the type I receptor of bone morphogenetic protein (BMP) signaling. Hou *et al.* discovered that exosomal miR-128-3p derived from NSCs significantly elevated myelin basic protein expression in OPCs while suppressing BMP signaling. Moreover, NSC-derived exosomal miR-128-3p conferred protection against fibrinogen-induced demyelination linked to BMP signaling, resulting in a decrease in infarct volume and improvement in neurological function following MCAO 85. In primate models, EVs treatment led to a reduction in damaged oligodendrocyte density and improved myelin maintenance. The study's findings were confirmed by observing an elevation in the expression of genes associated with myelin production and a rise in the number of oligodendrocytes actively involved in myelination within the white matter areas surrounding lesions. The enhancements in myelination were positively linked to the pace of motor function restoration, suggesting that improved maintenance of myelin contributes to the recuperation process following brain injury in older primates 84.

Interestingly, SC-EVs can improve mitochondrial biogenesis. Li *et al.* demonstrate that EVs derived from neural stem cells markedly enhance mitochondrial biogenesis by activating the sirtuin 1 (SIRT1) - peroxisome proliferator-activated receptor-γ coactivator-1ɑ (PGC1ɑ) signaling pathway and increasing the synthesis of nuclear respiratory factor 1 (NRF1) and cytochrome C oxidase IV (COXIV). Moreover, these EVs inhibit astrocyte activation, although they do not suppress amyloid-β production (Figure 2A) 62.

In conclusion, these studies underscore the promising therapeutic applications of both MSC-EVs and NSC-EVs in the treatment of neurological disorders. Both types of EVs offer similar therapeutic benefits, albeit through different combinations of mechanisms that promote neural repair, including neuroprotection, angiogenesis and preservation BBB integrity, alleviation of neuroinflammation, and other functions. However, the choice of the most appropriate SC-EVs for particular neurological diseases remains uncertain. While some studies have shown that NSC-EVs may be more effective than MSC-EVs, for instance, in a comparative analysis of the therapeutic impacts of NSC-EVs and MSC-EVs, it was determined that NSC-EVs were more effective. They demonstrated superior results in mitigating neural injury and bolstering the systemic immune system's response within a mouse model of thromboembolic stroke 98. This finding is supported by another study showing that NSC-EVs outperformed MSC-derived EVs in addressing neuroinflammation 99. Besides, in a head-to-head evaluation, the therapeutic efficacy of NPC-EVs was determined to be at least as effective as that of MSC-EVs. The benefits are clear, not just in terms of boosting cells' resistance to damage caused by a lack of oxygen in laboratory settings, but also in promoting the regeneration of brain tissue after a stroke and aiding in the recovery of neurological functions within a mouse model of stroke 69. Nevertheless, these findings should not be construed as indicating that MSC-EVs are inherently less advantageous than NSC-EVs. Therefore, further evidence and a better understanding of the underlying mechanisms are needed to facilitate the translation of these findings into clinical applications.

## 5. Engineered SC-EVs for therapies

**Figure 3:**
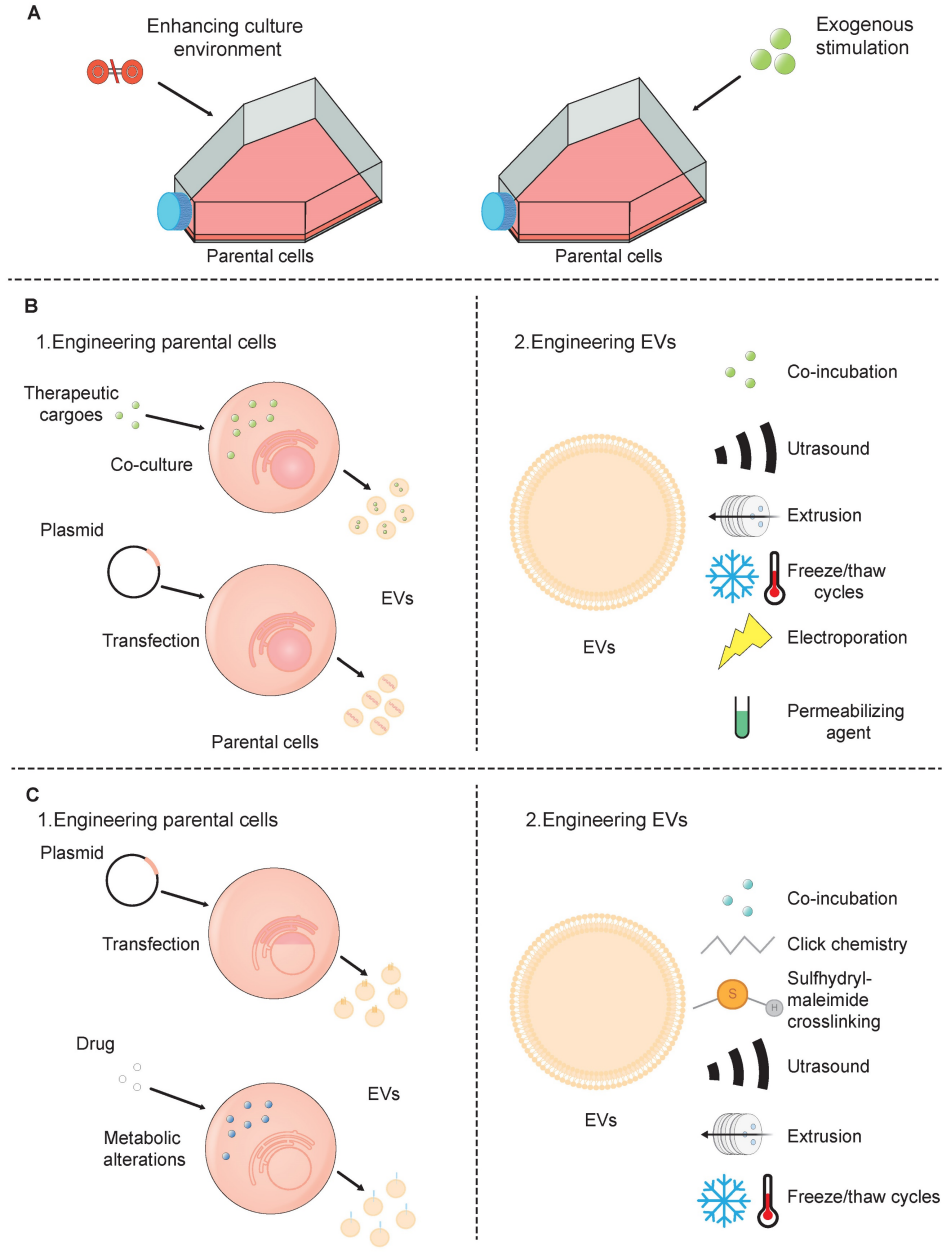
** Strategies for the engineering of SC-EVs.** (A) preconditioning of parental cells; (B) loading of therapeutic cargoes; and (C) surface modification.

Although SC-EVs have considerable promise as a therapeutic strategy, there are a few drawbacks such as low targeting efficiency, inconsistent treatment outcomes, and limited production yield 100. These challenges can be addressed through various engineering biotechnologies (Figure 3).

### 5.1 SC-EVs engineered techniques

#### 5.1.1 Preconditioned SC-EVs

The generation of SC-EVs is affected by the cell type and surrounding conditions, yet it can be enhanced through artificial means. There are two prevalent techniques employed for the preconditioning of SC-EVs: (1) improving the conditions within which cells are cultured, such as by employing three-dimensional (3D) culture techniques, and (2) applying external stimuli to the cells.

3D culture is a laboratory technique used to culture and study cells in an environment that more closely resembles the natural three-dimensional structure of tissues and organs within the human body. Some studies have demonstrated that MSCs within a hollow fiber bioreactor increased the total production of EVs by up to 19.4-fold in 3D culture 101, and when combined with tangential flow filtration (TFF), MSCs can enhance the yield of EVs by as much as 100-fold 102. Besides, stem cells can undergo preconditioning during cell culture to enhance the bioactivity of SC-EVs, such as hypoxia 27, 103, 104, proinflammatory cytokines 105, 106, Chemical stimuli 107, low electric currents 108, and collagen scaffolds 109. However, it is crucial to examine the contents generated within SC-EVs that could potentially have adverse effects on therapeutic outcomes because preconditioning can impose stress on parental cells.

#### 5.1.2 Drug-loaded SC-EVs

As cell engineering progresses, researchers now possess the capability to genetically program cells in a way that enables them to incorporate specific cargo into SC-EVs. This cargo includes a range of therapeutic agents such as peptides, proteins, chemical compounds, and nucleic acids. The current strategies for engineering SC-EVs can be broadly categorized into two directions: (1) pretreating the donor cells, followed by the isolation of drug-loaded SC-EVs from stem cells; and (2) directly loading drugs in isolated SC-EVs.

Strategies for the pretreatment of donor cells are typically classified into two primary types: (1) direct co-culture of donor cells with drugs; and (2) genetic engineering of donor cells. In the initial approach, drugs are cultivated in conjunction with the donor cells, leading to their inherent presence within the SC-EVs that are subsequently released 110. This technique is quite simple and straightforward, eliminating the need for further modifications of the SC-EVs. Nevertheless, the transfection efficiency obtained through this approach tends to be on the lower side, which limits its application to primarily chemical substances that have a low level of cytotoxicity 111. In the second method, donor cells are transfected with plasmid encoding genes of interest to express or overexpress specific therapeutic molecules. When these cells release SC-EVs, they carry the desired drug cargo, which can enable a continuous collection of SC-EVs, mitigate adverse impacts on molecules, maintain a biocompatible composition, and safeguard the physicochemical parameters of SC-EVs. However, its time-consuming and complex nature for establishing a producing cell line and its limitation in being applied primarily to SC-EVs derived from stem cells rather than isolated SC-EVs or those obtained from body fluids 112.

Directly loading drugs in SC-EVs can be delivered using three main approaches: (1) incubating SC-EVs with a drug or dye solution, which facilitates their permeation through diffusion or endocytic uptake. During the incubation of cargoes, SC-EVs successfully encapsulated various low molecular weight compounds 113. While this method is cost-effective and easy to execute, its efficiency is compromised due to the limited capacity within SC-EVs, the small size of their pores, and the hydrophobic properties of their lipid membranes, which collectively restrict drug penetration. (2) loading drugs into SC-EVs using physical strategies, including ultrasound, extrusion, freeze/thaw cycles, and electroporation. After being mixed with therapeutic drugs, ultrasound exposure can lead to either partial disruption of EV membrane integrity, allowing drug diffusion inward, or complete membrane disruption, leading to the release of EV contents. Subsequently, the membranes may self-assemble to encapsulate the drug solution used during sonication 114. EV-like nanovesicles, constructed from the plasma membrane of parent cells, are primarily generated by extrusion through a series of polycarbonate membrane filters with decreasing pore sizes 115. These EV-like nanovesicles address the constraints linked to low yield, thus providing a method for the extensive production of extracellular vesicles. The freeze-thaw technique for loading SC-EVs with drugs begins with combining the pharmaceutical agents with the SC-EVs at room temperature. This mixture is then subjected to rapid freezing, either at -80°C or by immersion in liquid nitrogen. After the freezing process, the sample is allowed to thaw back to room temperature. This technique leverages the temporary formation of ice crystals, resulting in the disruption of plasma membranes and significant structural and functional changes within the cell membranes. These changes include lateral phase separation, and membrane fusion 116. Consequently, this method can cause aggregation of EVs, leading to a heterogeneous distribution of EV sizes. Moving on to electroporation, this is a distinct technique that employs brief electrical pulses. These pulses create transient openings, or pores, in the plasma membrane 117. Through these pores, drugs or nucleotides can efficiently diffuse into SC-EVs. Electroporation is frequently utilized for the encapsulation of larger biomolecules, such as siRNA or miRNA, into SC-EVs. Nonetheless, certain research has suggested that this process may lead to the aggregation of EVs. Significantly, Johnsen *et al.* demonstrated that the inclusion of trehalose in the electroporation buffer could effectively maintain the structural integrity of SC-EVs and avert their aggregation 118. (3) loading drugs into SC-EVs using permeabilizing agents. For example, saponin, a type of surfactant molecule, has the ability to interact with cholesterol present in cell membranes. This interaction results in the formation of pores that facilitate an increase in the permeability of the membrane 119. By increasing permeability, saponin allows for the passage of various proteins into the SC-EVs, including the model enzyme catalase. Significantly, this method not only enhances the transport of proteins but also retains the structural integrity and enzymatic functionality of complexes like catalase 120.

The techniques used to incorporate molecules and pharmaceuticals into Small Extracellular Vesicles (SC-EVs) differ in their effectiveness. As previously noted, methods such as sonication and extrusion have been found to be the most efficient in terms of loading capacity within SC-EVs, outperforming other strategies like freeze-thaw cycles and passive incubation 116. However, it is important to consider the characteristics of the drugs and to find the most optimized techniques.

#### 5.1.3 Surface-modified SC-EVs

Surface modification enables the introduction of distinct functionalities into natural SC-EVs by targeting peptides or sites for chemical alteration through genetic manipulation 121. Much like the process of loading cargo, the process of surface functionalization of SC-EVs can be broadly categorized into two distinct approaches: (1) alteration of the donor cells, which is then followed by the extraction of the SC-EVs with the desired modifications from the stem cells; (2) alteration of the SC-EVs' surface, which is succeeded by the removal of any residual modifying agents to obtain a purified SC-EV preparation.

The genetic modification of donor cells is typically performed using plasmid vectors that carry genes for targeting ligands. These ligands are engineered to be fused with various types of transmembrane proteins 122. The genetic engineering of donor cells offers a final EV product free from chemical contaminants and with high specificity in modification. However, this approach comes with drawbacks. One significant challenge is the considerable effort involved in developing a genetically modified cell line that is capable of effectively generating the targeted molecules. Additionally, there is the possibility that the fusion of peptides with the targeting segment could interfere with the normal functions of the proteins present on the EV membrane. Moreover, donor cells can undergo metabolic alterations through endogenous synthesis or specific cleavage processes. For instance, SC-EVs with an elevated expression of mannose were produced by growing cells in an environment containing kifunensine, an inhibitor of mannosidase enzymes. This treatment leads to an increased accumulation of mannose residues on the glycoproteins present on the cell surface 123. However, the metabolic modification approach for obtaining modified SC-EVs has limitations, including a restricted range of applications and the inability to extend this method to isolated SC-EVs.

In contrast to the modification of donor cells, the methods of modifying the EV surface, including co-incubation, chemical, and physical strategies, are easier. SC-EVs can be directly modified by co-incubating them with specific peptides, which interact with the SC-EVs through non-covalent binding to their surface 124. The drawbacks of using peptides as targeting tools stem from their inherent instability, rendering them vulnerable to degradation or hydrolysis. To address the challenge of protease susceptibility, D-amino acid isomers can be incorporated into peptides that are more resistant to proteolytic degradation 125. Alternatively, the stability of peptides can be enhanced by introducing glycosylation sites within their sequence 126. When it comes to the chemical modification of EV surfaces, a common approach involves the covalent attachment of targeting agents to the amino groups present on the surface proteins of SC-EVs. A widely used technique for this purpose is click chemistry, which includes two main types: copper-catalyzed azide-alkyne cycloadditions (CuAAC) and strain-promoted alkyne-azide cycloadditions (SPAAC) 127. This method involves a two-step process: first, the EV proteins are chemically modified to introduce alkyne groups; second, these modified proteins are conjugated with an azide-containing molecule. The propensity of alkyl and azide groups to react and form triazole rings is superior to that of conventional cross-linking agents. This enhanced reactivity affords greater precision and control during the conjugation process at the specific target site 128, 129. Importantly, the conjugation process is designed to be non-disruptive to the fundamental properties of SC-EVs. It does not change the size or the cell-binding characteristics of the SC-EVs, ensuring that both their structural and functional integrity are maintained. Besides, sulfhydryl-maleimide crosslinking is also a promising approach. Sulfhydryl groups are ubiquitously found in the majority of proteins such as membrane proteins. As a result, SC-EVs can be equipped with a wide variety of functional molecules through the formation of a biocompatible bond between sulfhydryl groups and maleimide moieties 130. Physical methods include ultrasound, extrusion, and freeze-thaw cycling, as the above.

### 5.2 The applications of engineered SC-EVs

Enhancements to the therapeutic efficacy of SC-EVs could be achieved by improving their target recognition, biodistribution, and their ability to efficiently cross the BBB through manipulation and modification of the surface or content using engineered approaches, ultimately leading to improved treatment outcomes. SC-EVs have utility in AD, PD, and stroke (Table 3).

#### 5.2.1 The applications of engineered SC-EVs in AD

AD, a form of neurodegenerative disorder, is marked by a gradual decline in cognitive functions 151. In AD, the brain exhibits the presence of amyloid beta (Aβ) plaques, an overabundance of hyperphosphorylated Tau protein, and the formation of neurofibrillary tangles (NFTs). These aggregates interfere with the normal functioning of neurons, leading to cytotoxicity. The cytotoxicity arises from various disruptions, including the leakage of ions across cell membranes, irregularities in calcium levels, and the deterioration of the electrical potential of the membrane 152, 153. These processes result in neuronal apoptosis, synaptic degeneration, and a spectrum of cognitive and functional impairments, encompassing learning, behavioral, and motor deficits 154.

In recent years, an increasing body of evidence has underscored the therapeutic potential of engineered SC-EVs in AD. Preconditioning has been shown to enable EVs to modulate function and improve production. EVs sourced from MSCs that had been conditioned with the secretome of lipopolysaccharide (LPS)- or Aβ-activated microglia showed heightened potency in curbing neuroinflammation, reducing demyelination, and improving memory and anxiety-related behavioral impairments in murine models of neuroinflammatory conditions 131.

**Figure 4:**
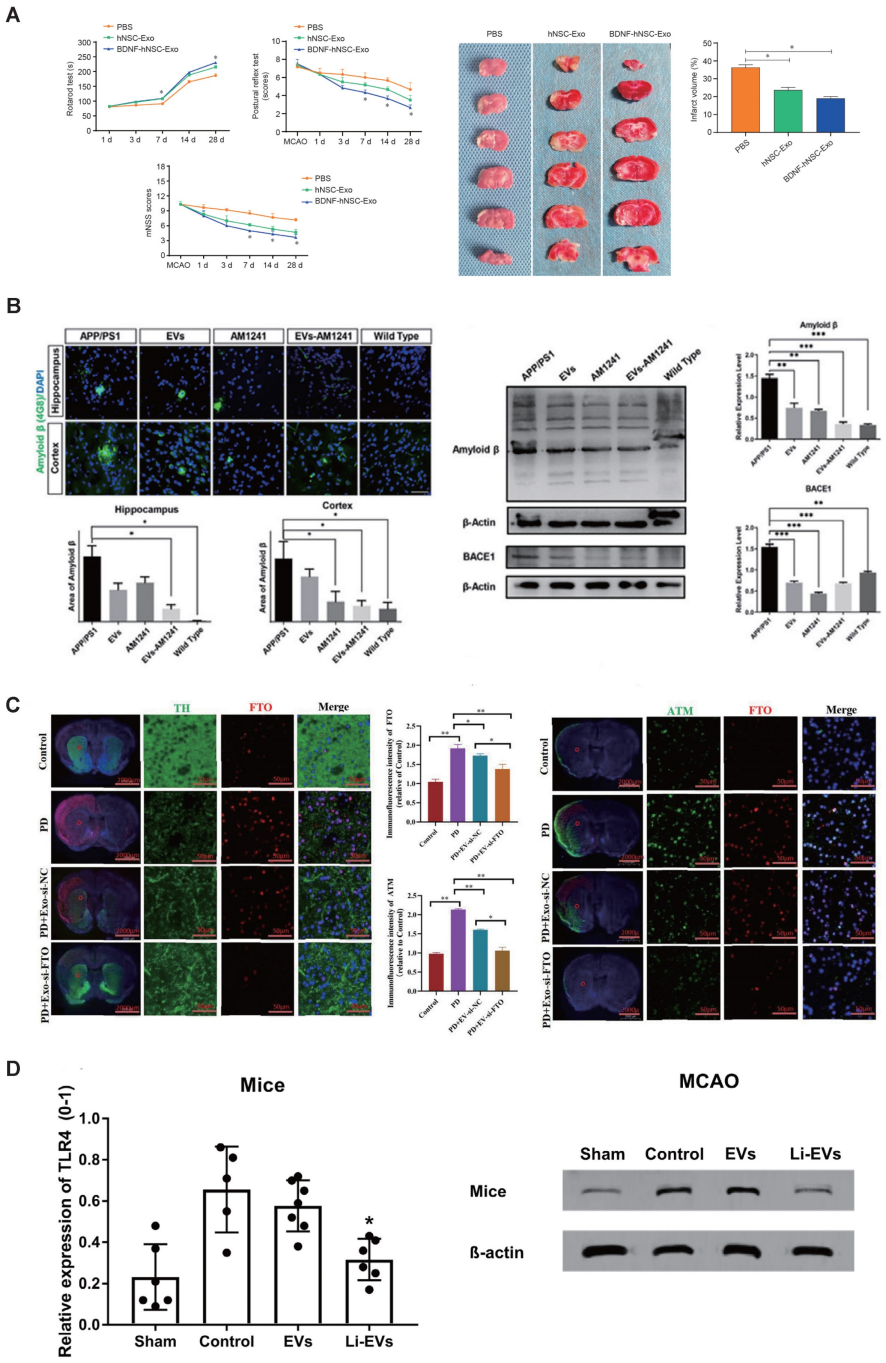
** The applications of engineered SC-EVs.** (A) BDNF-hNSC-Exo enhances neurological function and reduces brain damage in rats following cerebral ischemia. Adapted with permission from 141, copyright 2022 Medknow. (B) EVs-AM1241 alleviated the pathological neurodegenerative changes in AD mice 138, copyright 2023 ELSEVIER. (C) Synergistic loading of si-FTO into exosomes effectively protects against dopaminergic neuronal death in a Parkinson's disease model in vivo. Adapted with permission from 139, copyright 2023 BMC. (D) Li-EVs decrease the expression of TLR4 in mice. Adapted with permission from 135, copyright 2021 WILEY.

Besides, many EVs exert an effect by containing therapeutic drugs. For example, Zhu *et al.* employed biocompatible EVs derived from MSCs as carriers for delivering the CB2-targeted medication AM1241 (EVS-AM1241) to mitigate neurodegenerative progression. This intervention facilitated Aβ phagocytosis, fostered neurogenesis, and ultimately enhanced learning and memory in AD model mice. These effects were mediated through the calcium-Erk signaling pathway (Figure 4B) 138. Additionally, Xu *et al.* utilized lentivirus to infect MSCs with the Src homology 2 domain-containing protein tyrosine phosphatase-2 (SHP2) gene, thus obtaining MSC-EVs enriched with a high level of SHP2 (MSC-EVs-SHP2) in the context of AD. Consequently, MSC-EVs-SHP2 significantly induce mitophagy, leading to the improvement of mitochondrial damage-induced apoptosis and the inhibition of NLRP3 activation in neuronal cells 137. Mitophagy not only reduces neuronal cell apoptosis and neuroinflammation but also leads to the restoration of synaptic loss and the amelioration of cognitive decline in a mouse model of AD. Additionally, ADMSCs were transfected with a miRNA-22 mimic to produce miRNA-22-loaded EVs, utilized for the treatment and neural repair of AD. EV-miRNA-22 demonstrated the ability to enhance motor function, promote nerve cell survival, and reduce the expression of inflammatory factors in APP/PS1 mice. Moreover, EVs loaded with miRNA-22 have been observed to reduce the secretion of inflammatory cytokines in vitro by suppressing the process of pyroptosis 155.

Drawing from the enzymatic activity of membrane proteins found in MSC-EVs, Huang *et al.* have successfully engineered and created a hydrogel made up of self-assembling peptides, which are specifically designed to be degradable by enzymes present in cellular membranes. Upon intranasal delivery, this smart release hydrogel prolonged the retention of MSC-EVs at the site of administration, enabling a controlled release of MSC-EVs. This approach effectively mitigated neuronal damage, fostered neurogenesis, and ameliorated memory deficits in 5×FAD AD model mice 147. To improve siRNA delivery to brain neurons via nasal administration, Li *et al.* developed lesion-targeting EVs. These MSC-derived EVs were modified with the RVG peptide, which binds to neuronal acetylcholine receptors. Following intranasal delivery, these EVs penetrated the nasal mucosa, reaching afflicted brain regions. Within the cytoplasm, EV cores facilitated controlled siRNA release in the high-ROS environment, leading to decreased BACE1 and caspase-3 levels, thus reducing Aβ plaques and neuronal apoptosis. Additionally, MSC-derived EVs decreased reactive astrocyte numbers. Collectively, these strategies synergistically ameliorated AD pathologies and cognitive deficits 149.

These findings suggest a promising therapeutic approach that may help mitigate the symptoms of Alzheimer's disease. However, it is essential that additional clinical studies are conducted to fully assess the efficacy and safety of this strategy in a medical context.

#### 5.2.2 The applications of engineered SC-EVs in PD

PD, ranking as the second most prevalent chronic neurodegenerative condition globally 156, is distinguished by several key neuropathological features. These include a significant loss of dopaminergic (DA) neurons within the substantia nigra (SN) region of the brain, a consequent decrease in dopamine levels within the striatum, and the formation of intracellular aggregates composed of the ɑ-synuclein protein 157. The complex pathophysiology of PD stems from a convergence of multiple molecular pathways and cellular mechanisms. These include disruptions in the regulation of ɑ-synuclein protein homeostasis, impairments in mitochondrial function, increased oxidative stress, perturbations in calcium ion balance, deficits in axonal transport processes, and the detrimental effects of neuroinflammation 158. PD's defining symptoms encompass motor dysfunctions, such as bradykinesia, rigidity, tremors, and gait disturbances, in addition to non-motor manifestations such as pain, fatigue, depression, and cognitive deficits 159. Current therapeutic approaches for PD predominantly center on surgical interventions and a limited array of pharmaceutical options, while potential future treatments explore avenues like deep brain stimulation, transplantation of dopaminergic neurons derived from stem cells, and gene therapy as potential curative strategies 160, 161.

Recent research has demonstrated that the administration of specially engineered SC-EVs has led to significant improvements in a 6-hydroxydopamine (6-OHDA) induced mouse model of Parkinson's disease. These improvements include the reduction of neuronal cell apoptosis, amelioration of neurobehavioral deficits, suppression of neuroinflammation, decrease in oxidative stress, and the dispersion of abnormal protein aggregates. Notably, the therapeutic impact of EVs is primarily dependent on their contents, which can be modified to activate or suppress specific processes in target cells. For example, EV-encapsulated miR-181a-2-3p derived from MSCs could potentially mitigate oxidative stress in PD by enhancing SH-SY5Y cell proliferation and boosting superoxide dismutase (SOD) levels. Additionally, it suppresses apoptosis and reduces levels of malondialdehyde (MDA) and reactive oxygen species (ROS) by modulating the expression of growth-response-1 (EGR1) through the inhibition of NOX4/p38 mitogen-activated protein kinase (MAPK) signaling. This process helps prevent the apoptosis of dopamine neurons 143. Meanwhile, Li *et al.* also employed miR-188-3p-enriched EVs derived from ADMSCs to target cell division protein kinase 5 (CDK5) and NLRP3 in PD model, resulting in the restraint of autophagy and pyroptosis 144. Using si-FTO to suppress FTO expression simultaneously decreased the overproduction of α-synuclein and the underproduction of tyrosine hydroxylase, which helped to reduce neuronal death in PD models. Furthermore, EVs derived from MSCs effectively delivered si-FTO to the striatum of animal brains. This resulted in a significant reduction of α-synuclein levels, protection of dopaminergic neurons, and a recovery of tyrosine hydroxylase expression in PD mice brains (Figure 4C) 139. Interestingly, Peng *et al.* have developed a sophisticated self-targeting nanocarrier named PR-EXO/PP@Cur. This breakthrough technology merges the healing potential of extracellular vesicles derived from MSC-EVs with the powerful anti-inflammatory and therapeutic effects of curcumin. The PR-EXO/PP@Cur nanocarrier has shown remarkable capabilities, including the autonomous navigation through multiple cellular membranes and the targeted delivery of its cargo directly into the cytoplasm of specific cells. PR-EXO/PP@Cur effectively targets the complex pathologies of PD through a synergistic three-pronged strategy. By increasing the concentration of the therapeutic agent at the site of action, it reduces the accumulation of ɑ-synuclein aggregates, facilitates the restoration of neuronal function, and alleviates neuroinflammation 150.

Although SC-EVs hold promise as a strategy against PD through multiple mechanisms, translating preclinical findings to clinical studies poses significant challenges because none of the currently used animal models fully replicate all the defining features of PD. Developing more robust disease models is essential to gain a deeper understanding of the pathophysiology.

#### 5.2.3 The applications of engineered SC-EVs in stroke

Stroke remains a leading cause of both mortality and disability across the globe. Regrettably, to date, no universally effective treatment strategy has been established 162. It disrupts the typical operation of specific brain areas, resulting in enduring brain damage and impairments in both motor and cognitive functions 163. Ischemic stroke (IS), the most common form of stroke, is marked by an initial ischemic episode that leads to a lack of blood flow and oxygen supply to the brain tissue 164. Frequently, these obstructions are triggered by blood clots, which are the primary cause of ischemic strokes 165.

In contrast to the treatment strategies for neurodegenerative diseases, the primary therapeutic focus in stroke treatment is on mitigating neuroinflammation, enhancing neuroprotection, and fostering neuroregeneration. Literature suggests that the primary driver of neuronal damage in stroke cases is the inflammatory reaction initiated by glial cells 166. Over recent years, an increasing amount of research has underscored the promising therapeutic effects of SC-EVs that have been treated under hypoxic conditions. For example, Wu *et al.* demonstrated that the administration of hypoxia-treated EVs (Hypo-EVs) can lead to significant improvements in behavioral deficits and a reduction in the size of infarcted areas in a mouse model of stroke. This therapeutic effect is believed to be mediated by a specific regulatory pathway involving miR-214-3p, which is enriched in Hypo-EVs. The miR-214-3p pathway works by downregulating the PTEN/Akt signaling cascade, a process that is instrumental in enhancing the recovery of neurological function after ischemic and reperfusion injuries 132. EVs sourced from MSCs subjected to hypoxic conditions have shown a significant improvement in microvascular growth within ischemic tissue. This was evidenced by an increase in both microvascular length and branching point density, as observed using 3D light sheet microscopy over a period of up to 56 days. Furthermore, these EVs have been associated with a decrease in delayed neuronal degeneration and brain atrophy, which collectively contribute to improve neurological recovery 133. Besides, the findings revealed that infarct-preconditioned EVs, in comparison to normal EVs, significantly enhanced vascular remodeling and neurological function recovery post-stroke. The upregulation of specific miRNAs and their target genes associated with vascular smooth muscle cells underscored the crucial role of vascular remodeling in stroke recovery 134. Notably, Haupt *et al.* discovered that intravenous administration of EVs preconditioned with lithium led to enhanced neurological recovery and neuroregeneration for up to 3 months compared to controls and EV-treated mice. Specifically, Li-EVs exhibited significantly elevated levels of miR-1906, identified as a novel regulator of TLR4 signaling. Li-EVs effectively decreased posthypoxic and postischemic TLR4 levels, leading to inhibition of the NF‐κB signaling pathway, reduced proteasomal activity, and decreased expression of inducible NO synthase and cyclooxygenase-2. These collective effects resulted in diminished post-stroke cerebral inflammation (Figure 4D) 135.

Furthermore, the treatment of drug-loaded SC-EVs led to significant improvements functional levels in stroked models. Zhou *et al.* engineered MSCs to overexpress BDNF and purified the resulting EVs using anion exchange chromatography. In a mouse model of ischemic stroke, intranasally administered EVs were able to specifically target the region surrounding the infarct. The enrichment of EVs with BDNF substantially amplified their therapeutic potential, leading to marked improvements in behavioral functions and neural reparative processes. These enhancements included a decrease in the size of the infarct, stimulation of neurogenesis, promotion of angiogenesis, enhancement of synaptic plasticity, and the maintenance of nerve fibers. Additionally, this treatment reduced inflammatory cytokine expression and glial response. The intranasal delivery of both EVs and BDNF-loaded EVs upregulated genes associated with neuroprotection while downregulating inflammation-related genes. Additionally, BDNF-loaded EVs specifically activated the BDNF/TrkB signaling pathway in the ischemic brain 140. Similarly, hNSC-EVs loaded with BDNF after 24 hours of co-incubation displayed the capacity to enhance cell survival and induce differentiation. In a rat ischemic stroke model, BDNF-hNSC-EVs efficiently suppressed microglial expression and stimulated the differentiation of endogenous NSCs into neurons, consequently leading to a reduction in infarct volume and an enhancement of neurological function (Figure 4A) 141. Inspiringly, BMSC-derived EVs containing IncRNA KLF3-AS1 exhibited a reduction in cerebral infarction and improvement in neurological function in MCAO mice by enhancing Sirt1 deubiquitinating. Mechanistically, KLF3-AS1 inhibited the ubiquitination of Sirt1 protein by inducing USP22 expression.

Furthermore, KLF3-AS1 acted as a sponge for miR-206, leading to the upregulation of USP22 expression 142. Moreover, Xin *et al.* discovered that the improved neuro-functional recovery observed in stroke cases, as a result of miR-17-92 cluster-enriched MSC EVs, can be linked to an augmentation in axonal extension and myelination. The activation of the PI3K/Akt/mTOR pathway, which is thought to be a consequence of PTEN downregulation, is likely responsible for facilitating these beneficial effects on neural repair and function 145. In MCAO mice, both NPC-EVs and endothelial progenitor cell-derived EVs (EPC-EVs) were found to decrease infarct volume and neurological deficits, reduce neural apoptosis and ROS production, and enhance dendritic spine density. These effects were found to be connected to the reduction in Nox2 expression and the upregulation of BDNF, along with the phosphorylation of its receptor, known as p-TrkB/TrkB. Notably, the simultaneous use of NPC-EVs and EPC-EVs demonstrated a synergistic effect, yielding superior results compared to the use of either type of EVs in isolation. Furthermore, the synergistic effects of EPC-EVs-miR-126 + NPC-EVs-miR-210 were superior to those of NPC-EVs + EPC-EVs 146.

In addition, engineered EVs can also improve the efficiency of therapy by surface modifications. Recently, Tian *et al.* have shown that through intravenous injection, RGD-C1C2-bound EVs can specifically target the affected area in ischemic brain injury. This protein has a high affinity for self-assembly onto the surface of the EVs, facilitating the precise homing of RGD-EVs to the lesion site. Consequently, they strongly suppressed the inflammatory response by inhibiting the MAPK pathway, which is an inflammation-related pathway 33.

Additionally, a hyaluronic acid (HA) hydrogel modified with catechol can encapsulate NSC-derived EVs while preserving their activity 167. This hydrogel mimics the 3D microenvironment of tissues, promoting endogenous cell adhesion and proliferation in ischemic regions. The controlled release of NSC-derived EVs from the adhesive HA hydrogel shows promise in enhancing cerebral angiogenesis and improving neurological function post-ischemic stroke 148.

In conclusion, these findings suggest a strategy that could potentially reduce infarct volume and provide implications for alternative therapeutic approaches to stroke. However, future preclinical studies on the therapeutic potential of SC-EVs in stroke should focus more on gaining a comprehensive understanding of the mechanisms of action of EVs.

## 6. Route of administration

Discussion about the administration route of SC-EVs-based therapy is increasing in recent years, including intravenous injection, intracerebral injection, intraventricular injection, and intranasally.

Previous research has suggested that intravenous administrations of SC-EVs can ameliorate neurological impairments. In a comparative analysis between femoral vein and retroorbital injection as delivery routes, the study observed a consistent biodistribution pattern for NPC-EVs under both ischemic and non-ischemic conditions. NPC-EVs were found in peripheral organs like the liver and lungs, in addition to their presence in the brain. Nevertheless, the liver and lungs exhibited a higher concentration of these EVs compared to the brain 69. As a result, to counteract the tendency of SC-EVs to accumulate in the liver, spleen, gastrointestinal tract, and lungs after intravenous injection, higher doses are required. Notably, administering EVs via the tail vein and lateral ventricle led to improved behavior in PD model rats, with no significant difference observed between the two injection techniques 64. However, in the case of AD-like behavioral performance enhancement in mice injected with streptozotocin, the administration of BMSC-EVs via the lateral ventricle, rather than through the tail vein, has shown better outcomes 58. Nonetheless, it's essential to acknowledge that intracerebral and intraventricular injections are highly invasive and have low compliance.

In recent years, the intranasal (IN) route has garnered increasing attention due to its distinctive transport pattern 168, 169, which presents a compelling method for the direct and non-invasive introduction of pharmaceuticals or cellular therapies into the brain. The nasal cavity is equipped with olfactory sensory neurons in the olfactory region and branches of the trigeminal nerve in the respiratory region, both of which have exposed nerve terminals under the mucosal layer. This arrangement allows drugs to penetrate the epithelial layer and be transported to the olfactory bulb or pons along the axons of these neurons. From there, the drugs can enter the perivascular space of the brain's blood vessels or spread through the cerebrospinal fluid, leading to a broad distribution across the central nervous system. This route of administration bypasses the digestive system and liver metabolism, which can improve drug absorption and decrease the amount needed to reach therapeutic levels 170. Pharmacokinetic research has shown that although the nasal epithelium may reduce the bioavailability of drugs delivered intranasally, the concentration of the drug in most central nervous system regions is roughly tenfold higher following intranasal administration compared to systemic injection when blood drug levels are the same 171. Moreover, the non-invasive characteristic of intranasal delivery is a significant benefit. Devices for intranasal delivery, such as sprays, atomizers, nasal drops, or other similar tools, offer simple application methods and minimize the risk of harm to patients 172.

In an ETH1 stroke model, the use of glucose-coated gold nanoparticles (GNPs) for labeling, in conjunction with CT imaging, revealed that the IN route resulted in better accumulation in the brain's injured area compared to the intravenous route 173. Zhou *et al.* found that intense fluorescence was observed surrounding the periinfarct region of the brain after the intranasal administration of EVs, with minimal signal detected in other organs such as the heart, lung, liver, spleen, and kidney, suggesting a targeted delivery of EVs to the lesion area. Furthermore, the nasal mucosa structure resembled that of the PBS group, exhibiting no apparent abnormalities, thus indicating the absence of mucosal toxicity associated with the intranasal delivery of BDNF-EVs in mice 140. Rohden *et al.* illustrated that intranasal injection of EVs 24 hours after ischemic stroke in rats led to enhancements in EV quantity, blood-brain barrier integrity, and vascular stabilization within the recoverable peri-infarct zone 74. Losurdo *et al.* conducted a study where they utilized intranasal delivery of EVs sourced from cytokine-preconditioned MSCs to provide neuroprotective and immunomodulatory effects in 3xTg AD mice. This therapeutic strategy effectively reduced the activation of microglial cells, increased the density of dendritic spines, and regulated the inflammatory response in the treated mice 105.

Nevertheless, the primary concentration of ongoing preclinical studies is on substantiating the therapeutic efficacy of IN delivery, particularly in the context of EV-based treatments for CNS disorders. A majority of intranasal delivery research is performed on animal models, particularly rodents, whose anatomical structures and olfactory regions differ significantly from those of humans 174. Future investigations may need to include experiments on large animals, considering the substantial differences between human and rodent nasal cavities. Experimentation on large animal models is crucial for gaining insights into how different formulations affect the pharmacokinetics of a drug. Such studies in larger animals provide data that are more closely aligned with the pharmacokinetic profiles observed in humans, offering valuable and relevant information for translational research. Additionally, efforts in biotechnology are necessary to prolong the residence time of drugs within the nasal cavity, thereby increasing bioavailability 149, 150. Integrating EVs into formulations such as hydrogels could extend mucus retention and enhance bioavailability. Moreover, the development of appropriate formulations can enhance the compatibility of EVs with IN delivery systems. This optimization not only streamlines the administration process but also enhances the overall efficiency of drug delivery 147. Therefore, there is still considerable room for improvement in the IN delivery of EVs. Despite challenges, intranasal delivery offers significant promise and could become a pivotal element in the clinical application of therapies based on EVs.

## 7. The challenges and prospects of clinical translation

### 7.1 Clinical studies of SC-EVs

Over recent years, an expanding pool of research has underscored the promising therapeutic effects of EVs that originate from stem cells. This evidence has fostered a perception that EV-based therapies may offer superior safety and a wider range of applications compared to traditional cell therapy methods, even though clinical data have yet to be fully realized. A number of studies focused on the utilization of EVs or exosomes are registered on https://beta.clinicaltrials.gov (accessed on 18 October 2023), including NCT05490173, NCT05370105, NCT05326724, NCT05035134, NCT04928534, NCT04388982, NCT04202770, NCT03384433, NCT01860118. However, a large proportion of those are observational studies or not applicable. Notably, only two studies are in phase 2. They are the clinical trial NCT04388982 and NCT03384433. The registered clinical trial NCT04388982 investigates the safety and efficacy of EVs derived from allogenic adipose MSCs in the treatment of mild to moderate dementia caused by AD. While NCT03384433 evaluates improving patients with acute ischemic stroke who received miR-124-Loaded EVs derived MSCs.

Although the number of currently registered clinical studies is relatively limited, there is a noticeable increase in interest in these therapies. Looking ahead, it is anticipated that an increase in clinical studies will pave the way for the emergence of EV-based drugs on the market.

### 7.2 The challenges of SC-EVs therapeutic strategies

In recent decades, significant strides have been made in comprehending the biological characteristics and exploring potential therapeutic uses of SC-EVs in the realm of neurological disorders. However, there are several challenges of clinical translation to overcome.

Initially, the heterogeneity of SC-EVs is shaped by numerous factors, including donors and tissue origins, cell compositions, culture environments, diverse batches, methods of isolation and purification, as well as storage conditions of EVs. Variances in donors' health status, genetic makeup, gender, and age contribute to these diversities 175. Subsequently, tissues derived from different sources manifest distinct attributes, further contributing to this heterogeneity. Li *et al.* extensively examined the heterogeneous landscape of MSC-EVs, focusing on BMSC-EVs, UCMSC-EVs, and ADMSC-EVs, which are among the most intensively utilized. Their findings revealed that UCMSC-EVs exhibited superior drug loading and delivery capabilities, thereby offering promising prospects for future translational applications 176. Similarly, the culturing environment significantly influences SC-EVs yield and function, including factors such as the use of artificial preconditioning, various culture methods, and specific culture conditions 134. There is heterogeneity in surface markers, biophysical properties, and content among different batches of SC-EVs. Moreover, differences in EV isolation and purification methods, along with variations in EVs storage conditions, can alter the composition of SC-EVs. Simultaneously, the characterization methods and detection techniques for SC-EVs are not yet standardized. Therefore, it is crucial to establish standardized protocols for the cell source, production process, and content of SC-EVs. This standardization is essential for reducing the side effects associated with the variability of SC-EVs and for guaranteeing their consistency and reproducibility in therapeutic applications 177. Recent studies have elucidated some established physicochemical properties of EVs. Morphological examination of EVs can be conducted using transmission electron microscopy (TEM), which provides detailed imaging. Meanwhile, the particle size and concentration can be precisely measured with the Flow NanoAnalyzer 178. For protein content analysis, the BCA protein assay kit 47 offers a reliable quantitative method. Furthermore, the presence and quantity of cytosolic proteins derived from the parent cells, as well as specific EV markers like CD9, CD63, and CD81, can be assessed through flow cytometry. With the development of EV analysis techniques and the continuous deepening of research and understanding of EVs, it is necessary to establish quantitative and specific indicators for EVs from different stem cell sources, thus improving the quality standards of SC-EVs.

Then, the yield of SC-EVs is indeed a significant challenge in the clinical translation of EV-based therapies and diagnostics. Identifying methodologies that yield high quantities and are effective in producing and purifying SC-EVs is crucial. It is necessary to continually refine SC-EVs isolation techniques to optimize both yield and purity. The ultimate goal is to establish a standardized and uniform SC-EVs manufacturing process that adheres to current Good Manufacturing Practices (GMP). SCs are typically cultured in Petri dishes, or culture bottles, limiting the surface area for cell growth. The multi-layer cell factory cultivation process enables the medium-scale expansion and cultivation of SCs. However, this method requires a significant amount of manpower and space, and cells may be unevenly distributed across the layers. Therefore, it is indispensable to achieve highly repeatable cell cultivation and collection of cell supernatant by using a large-scale automated cell culture device. Bioreactors are essential tools for the large-scale production of extracellular vesicles due to their capability for dynamic monitoring. The hollow fiber bioreactor system, in particular, offers a dynamic environment that is conducive to cell cultivation. It also features a continuous medium collection system, which is vital for the efficient collection of the produced medium 179. A stirred-tank bioreactor can be used for the suspension cultivation of stem cells or their attachment to microcarriers suspended in the stirred vessel. The choice of suitable microcarriers is crucial for ensuring both the quantity and quality of cells, and it is closely related to the quality of SC-EVs collection and purification. Hollow fiber bioreactors allow stem cells to adhere directly to the coated surface of hollow fibers. Nonetheless, stem cells are known to be mechanically responsive, undergoing changes in their phenotype as a reaction to shear stress, cell clustering, and variations in the substrate they encounter within the dynamic environment of a 3D bioreactor 176. Thus, during the adherent cell culture process, real-time monitoring of cell proliferation status and quality is challenging. Son *et al.* discovered that culturing MSCs in a 3D spheroid within a micro-patterned well led to enhanced EV production. Compared to traditional 2D culture techniques, the EVs derived from the 3D culture platform, in terms of particle number, size, and purity, showed higher consistency not only across multiple batches from the same donor but also among different donors 180. Similarly, Han *et al.* developed a gelatin methacryloyl (GelMA) hydrogel for the 3D culture of MSCs. Their work suggests that a 3D environment can mimic the natural biological niches of MSCs, which significantly boosts the stem-like properties of these cells and, in turn, enhances the yield of MSC-derived exosomes 181.

Furthermore, maintaining the stability of SC-EVs during storage is crucial prior to clinical translation. The stability of stored extracellular SC-EVs is impacted by several factors, such as storage conditions, temperature, choice of storage solution, storage duration, and the occurrence of freeze-thaw cycles. Therefore, it is imperative to store SC-EVs in an environment not exceeding -80°C, while also avoiding repeated freeze-thaw cycles or significant temperature fluctuations 182.

Finally, assessing the safety of EV-based therapies is crucial. Potential adverse effects, immunogenicity, and off-target effects must be thoroughly investigated in preclinical studies and during clinical trials.

## 8. Prospects and conclusions

SC-EVs continue to hold promise as versatile agents, given their capacity to encapsulate abundant cargo, traverse the BBB, and exhibit high biocompatibility and low immunogenicity, coupled with their ease of engineering for modifications. The progress of SC-EVs in the realm of neurogenesis undeniably heralds a new era in the treatment of neurological diseases, potentially opening the door to clinical applications for nerve damage repair in the future.

However, several avenues require further exploration, including the identification of the optimal source of SC-EVs derived from stem cells exhibiting neurotherapeutic behavior, elucidating the neuroprotective properties of proteins or miRNA cargo within SC-EVs, and investigating molecular mechanisms such as the cargo selection process and the methods through which cells uptake SC-EVs. As technology continues to advance, we can expect to deeper exploration of the biologically active mechanisms and key components of extracellular vesicles. High-throughput techniques, such as single-cell transcriptomics and proteomics, have the potential to intricately unravel the molecular composition and dynamic changes of extracellular vesicles. Moreover, dynamic simulation and big data machine learning will play pivotal roles in simulating the release, transmission, and interaction processes of extracellular vesicles across various microenvironments. This not only aids in predicting the interactions between extracellular vesicles and recipient cells but also provides targeted experimental design and more efficient data interpretation. Simultaneously, methods based on big data and machine learning can harness large-scale clinical data to gain a deeper understanding of the biomolecular characteristics of extracellular vesicles, thereby fostering the development of personalized medicine that provides clinicians with more precise disease diagnoses and treatment plans. In the future, attention to surface modifications of extracellular vesicles aiming to enhance the targeted delivery of nucleic acids may be a focus. Moreover, considering the integration of CRISPR technology holds promise for achieving even greater precision in gene editing and therapeutic outcomes.

In this review, we provide a comprehensive overview of the methods employed to obtain SC-EVs, encompassing the isolation and differentiation of stem cells, and the isolation and purification of extracellular vesicles. Subsequently, we delve into an exploration of the inherent functions of native SC-EVs. Under physiological conditions, EVs play a pivotal role in neuroprotection, angiogenesis and preservation BBB integrity, alleviation of neuroinflammation, and other functions. Moving forward, we examine strategies for engineering SC-EVs to enhance their therapeutic effects and yield. This leads us to a focused discussion on their applications in AD, PD, and stroke. Finally, we describe the challenges associated with these extracellular vesicles in clinical translation and propose potential solutions.

## Figures and Tables

**Table 1:** The methods used for SC-EVs isolation and purification

Methods	Principle	Advantages	Disadvantages	References
**Differential Ultracentrifugation**	Differential Centrifugation of particles based on their size and density	High purity; Most commonly used method	Time-consuming; Damage or aggregation of EV	44
**Density Gradient Ultracentrifugation**	Specific density layers based on their size and density	High purity; Avoiding exosomal damage	Time-consuming; Complex operation; Limited sample volume	45
**Size-Exclusion Chromatography**	Based on hydrodynamic Radius	High yield; Preserves EV integrity; Fast; low-cost	Contamination with other particles of similar size	46
**Ultrafiltration**	Based on their size using membranes with specific pore sizes	High yield; High purity; Simple operation; Not require specialized equipment	Contamination; Low specificity	47
**Polymer Precipitation**	Precipitation by PEG	Fast; Simple; Not require specialized equipment	Relatively impure product; Low sample recovery	48
**Immunoaffinity chromatography**	Antibodies or aptamers that specifically bind to surface markers on EVs	High specificity; high sensitivity	Limited by availability of specific antibodies; High cost	49
**Microfluidic Isolation**	Based on various physical and chemical properties	Precise control and automation; Fast	Expensive equipment; Limited throughput for large samples	50

**Table 2:** Functions of native SC-EVs

Disease	Source of EVs	Animal Model	Administration Route	Therapeutic Effects	Mechanism of Action	Reference
**AD**	hUCMSCs	APP/PS1 mice	tail vein	↑cognitive impairments	↓Nrf2	57
				↓hippocampal Aβ aggregation		
				↓neuronal loss		
	mBMSCs	STZ mice	lateral ventricle	↑behaviors function	↑synapse-related proteins	58
			tail vein	↓hyperactivation of microglia and astrocytes	↑BDNF	
				↓imflammation		
	hiMSCs	STZ mice	intracisternal	↓neuroinflammation	containing miR-223-3p	59
				↓amyloid deposition	↓NLRP3/GSDMD	
				↓neuronal apoptosis		
				↓cognitive dysfunction		
	rBMSCs	Aβ_1-42_-injected rats	lateral ventricle	↑cognitive function	containing miR-29c-3p	60
				↓Aβ plaques, Aβ deposition areas and levels of Aβ_1-42_-injected	↓BACE1	
				↑NEP and IDE	↑Wnt/β-catenin	
				↓inflammatory cytokine		
	hBMSCs	5XFAD mice	intranasally	↑cognitive function	NA	61
				↓Aβ plaque		
	mNSCs	APP/PS1 mice	lateral ventricle	↑cognitive behavior	↑SIRT1	62
				↑mitochondrial biogenesis	↑PGC1c	
				↓astrocyte activation	↑NRF1	
					↑COXIV	
**PD**	TMSCs	MPTP mice	intraperitoneally	↓the loss of DA neurons	containing miR-100-5p	63
				↑nigro-striatal system function	↓NOX4	
				↑moter function	↓ROS	
				↓oxidative stress	↑Nrf2	
	hUCMSCs	6-OHDA rats	tail vein	↑moter function	contain MiR-7, miR-125-5p, miR-122-5p, miR-126-3p, miR-199-3p	64
			lateral ventricle	↑dopamine content		
				↓neuronal damage		
				↓microglial activation		
	hBMECs	MPTP mice	intraperitoneally	↑angiogenesis	↑ICAM1-SMAD3/P38MAPK	65
	hNSCs	6-OHDA mice	intracerebral	↑neuroprotection	containing hsa-mir-182-5p, hsa-mir-183-5p, hsa-mir-9, hsa-let-7	66
				↓dopaminergic neuronal loss	↓ROS	
				↓pro-inflammatory cytokines	↓associated apoptotic pathways	
**stroke**	rADMSCs	MCAO rats	lateral ventricle	↓brain injury	containing miR-22-3p	67
				↑neuron viability	↓KDM6B	
				↓apoptosis	↓BMP2/BMF axis	
	mBMSCs	MCAO mice	tail vein	↓infarct area	containing KLF4	68
				↓neuronal injury	↑lncRNA-ZFAS1	
				↓apoptosis	↓Drp1 m6A modification by targeting FTO	
	mNPCs	MCAO mice	femoral vein	↑neurological recovery	NA	69
	mADMSCs		retroorbital	↑neuroprotection		
				↑cell proliferation		
				↓pro-inflammatory		
	rBMSCs	MCAO mice	tail vein	↓infarct volume	↑miR-21-5p	70
				↑neurological functions	↑VEGF, VEGFR2, Ang-1, and Tie-2	
				↑microvessel density		
	BMSCs	MCAO rats	tail vein	↓cerebral infarction	↑ZO-1	71
				↓BBB leakage	↑Occludin	
				↓neurological function deficits	↓MMP activity	
					↓Caveolin-1	
					↓CD147	
					↓VEGFR2	
					↓VEGFA	
	hBMSCs	MCAO rats	tail vein	↓infarct volum	NA	72
				↓motor-coordination deficits		
				↓macrophage infiltrates		
				↑angiogenesis		
				↑neurogenesis		
	BMSCs	pMCAO rats	tail vein	↓cerebral infarction volume	↓Cav-1	73
				↓BBB permeability	↑ZO-1	
				↑neurological function	↑Claudin-5	
	hADMSC	MCAO rats	intranasally	↓infarct volume	NA	74
				↑long-term motor		
				↑behavioral impairment		
	hUCMSCs	MCAO mice	tail vein	↓ tPA-induced disruption of BBB integrity	miR-125b-5p targete TLR4	75
				↓ hemorrhage	↓NF-KB signaling in astrocytes	
				↓ astrocyte activation and inflammation		
	mNPCs	MCAO mice	femoral vein	↑poststroke BBB integrity	↓ABCB1 and MMP-9 regulation	76
				↓inflammatory cell recruitment	↓NF-κB pathway	
	mBMSCs	tMCAO P9 mice	ventricle	↓injury volume	NA	77
			intranasally	↓cytokine/ chemokine accumulation		
	hBMSCs	MCAO rats	tail vein	↓infarct volume	↑IL-33	78
				↑neurological function	↓ST2	
				↓neuronal deat		
	hBMSCs	MCAO young mice	intravenously	↓neurological deficits	NA	79
		MCAO aged mice		↓infarct volume		
				↓brain edema		
				↓neuronal injury		
				↑anti-inflammation		
				↓leukocyte infiltrate		
				↓monocytes and activated T cells		
	rBMSCs	MCAO rats	tail vein	↓brain infarct area	↓NLRP3 inflammasome-related proteins	80
				↓brain water content	↓pyroptosis-related proteins	
				↑neurological function		
				↑M1-polarized microglia shifting toward M2 phenotype		
	hUCMSCs	MCAO mice	tail vein	↓infarct volume	containing miR-146a-5p	81
				↓behavioral deficits	↓IRAK1/TRAF6 pathway	
				↓microglia activation		
				↓neuroinflammation		
	hiNSCs	MCAO mice	lateral ventricle	↓inflammatory response	containing hsa-miR-30a-5p	82
				↓oxidative stress	containing hsa-miR-7-5p	
				↑NSCs differentiation		
				↓cerebral infarction		
				↓neuronal death		
				↓glial scarring		
				↑recovery of motor function		
	rNSCs	MCAO/R Rat	tail vein	↓the infarction of brain tissues	carrying YBX1	83
				↓neuronal pyroptosis	↑stability of m6A-modified GPR30 by interacting with IGF2BP1	
					↑GPR30	
					↓activation of NLRP3 inflammasome through promoting NLRP3 ubiquitination by SPOP	
	BMSCs of young monkey	cortical injury aged monkeys	intravenous	↑functional recovery	↑myelin-related genes	84
				↓damaged oligodendrocytes	↑actively myelinating oligodendrocytes in sublesional white matter	
				↑myelin maintenance		
	mNSCs	MCAO mice	left stratum	↓infarct volume	containing miR-128-3p	85
				↓neurological function	↑myelin basic protein expression	
				↑OPC differentiation	↓BMP signaling	

AD, Alzheimer's Disease; PD, Parkinson's Disease; h, human; r, rat; m, mouse; BMSCs, bone marrow-derived mesenchymal stem cells; UCMSCs, Umbilical cord mesenchymal stem cells; ADMSCs, adipose-derived stem cells; iMSCs, induced pluripotent stem cell-derived mesenchymal stem cells; NSCs, neural stem cells; T-MSCs, trophoblast stage-derived mesenchymal stem cells; NPCs, neural progenitor cells; iNSCs, induced pluripotent stem cell-derived neural stem cells; 5XFAD mice, 5 familial Alzheimer's disease mutations; MCAO, middle cerebral artery occlusion; pMCAO, permanent middle cerebral artery occlusion; 6-OHDA, 6-hydroxy-dopamine; Nrf2, nuclear factor E2-related factor 2; STZ, streptozotocin; BDNF, brain-derived neurotrophic factor; Aβ, amyloid β; BACE1, β-site amyloid precursor protein cleaving enzyme 1; SIRT1, sirtuin 1; PGC1c, peroxisome proliferator-activated receptor-γ coactivator-1ɑ; NRF1, nuclear respiratory factor 1; COXIV, cytochrome C oxidase IV; ROS, reactive oxygen species; NOX4, nicotinamide adenine dinucleotide phosphate oxidase 4; BMP2, Bone morphogenetic protein 2; BMF, Bcl-2 modifying factor; VEGF, vascular endothelial growth factor; VEGFR, vascular endothelial growth factor receptor; MMP, matrix metalloproteinase; Cav-1, Caveolin-1; BBB, blood-brain barrier; ABCB1, ATP-binding cassette transporter B1; MMP-9, matrix metalloproteinase 9; NF-κB, nuclear factor-kappa B; IL, interleukin; ST2, suppression of tumorigenicity 2 receptor; NLRP3, NACHT, LRR and PYD domain-containing protein 3; IRAK1, interleukin-1 receptor-associated kinase 1; TRAF6, TNF receptor-associated factor 6; YBX1, Y box binding protein; GPR30, G protein-coupled receptor 30; SPOP, speckle-type POZ protein; BMP, bone morphogenetic protein; OPCs, oligodendrocyte progenitor cells; MAPK, mitogen-activated kinase; KLF4, Kruuppel-like factor 4; ZFAS1, zinc finger antisense 1; FTO, targeting obe- sity-associated protein; NA, not available.

**Table 3:** Applications of engineered SC-EVs

	Methodology	Source of EVs	Animal Model	Administration Route	Therapeutic Effects	Mechanism of Action	Reference
**Pre-condition**	secretome of lipopolysaccharide or amyloidbeta activated microglia	hMSCs	5xFAD mice	intranasally	↓microglia and astrocyte activation	↑miRNAs target key genes on the TLR4 signaling pathway	131
					↓amyloid deposition		
					↓demyelination		
					↓memory loss and motor and anxiety-like behavioral dysfunction		
	hypoxic	mBMSCs	MCAO mice	intravenously	↓infarct areas	↑miR-214-3p	132
					↓behavioral deficits	↓PTEN/Akt signaling	
	hypoxic	hBMSCs	MCAO mice	tail vein	↑microvascular length and branching point density	↑miR-126-3p, miR-140-5p, let-7c-5p	133
					↓neuronal degeneration	↓miR-186-5p, miR-370-3p, miR-409-3p	
					↓brain atrophy	52 proteins differentially abundant	
					↑neurological recovery		
	cerebral infarct tissue extracts	hUCMSCs	MCAO rats	tail vein	↑vascular remodeling	↑miRNAs and their target genes which is beneficial to vascular smooth muscle	134
					↑neurological function		
	lithium	mBMSCs	MCAO mice	femoral vein	↑neurological recovery	↑miR-1906	135
				retroorbital	↑neuroregeneration	↓TLR4	
					↓inflammation	↓NF‐κB	
						↓proteasomal activity	
						↓inducible NO synthase	
						↓cyclooxygenase-2 expression	
	BHD	rNSCs	MCAO rats	tail vein	↑neurological recovery	↑miR-124-5p miR-9a-5p miR-137-5p miR-184	136
					↑NSCs proliferation and differentiation		
**Drug-loading**	SHP2	mBMSCs	Aβ_1-42_-injected mice	intravenously	↓synaptic loss	↑mitochondrial damage-induced apoptosis	137
					↓cognitive decline	↓NLRP3 inflammasome	
					↓neuronal cells apoptosis		
					↓neuroinflammation		
	CB2 receptor agonist AM1241	mBMSCs	APP/PS1 mice	tail vein	↑learning and memory	↓calcium-Erk signaling pathway	138
					↓neuronal apoptosis		
					↑neuronal regeneration		
	FTO-targeted siRNAs	hUCMSCs	MPTP mice	intravenously	↓neuronal death	↑TH	139
					↓ɑ-Syn		
	BDNF	hiMSCs	MCAO mice	intranasally	↑functional behavior	↑neuroprotection-related genes	140
					↑neural repair	↓inflammation-related genes	
					↓infarct volume reduction	↑BDNF/TrkB signaling	
					↑neurogenesis		
					↑angiogenesis		
					↑synaptic plasticity		
					↑fiber preservation		
					↓inflammatory-cytokine expression		
					↓glial response		
	BDNF	hNSCs	MCAO rats	striatum of the ischemic hemisphere	↓infarct volume	NA	141
					↑neurological function		
					↓activation of microglia		
					↑differentiation of endogenous NSCs into neurons		
	IncRNA KLF3-AS1	mBMSC	MCAO mice	intravenously	↓cerebral infarction	↑Sirt1 deubiquitinating	142
					↑neurological function	↓miR-206	
					↑cell viability	↑USP22	
					↓apoptosis		
					↓inflammatory injury		
					↓ROS production		
					↓inflammatory factors		
					↓activation of microglia		
	miR-181a-2-3p	mADMSCs	6-OHDA mice	tail vein	↓apoptosis	↓EGR1	143
					↓oxidative stress	↓NOX4/p38 MAPK	
					↓ɑ-syn		
					↓4-HNE		
	miR-188-3p	mADMSCs	MPTP mice	intravenously	↓autophagy	↓CDK5	144
					↓pyroptosis	↓NLRP3	
					↑proliferation		
	miR-17-92	rBMSCs	MCAO rats	intravenously	↑neuro-functional recovery	↓PTEN	145
					↑axonal extension and myelination	↑PI3K/Akt/mTOR	
					↑axon-myelin remodeling		
					↑electrophysiological recovery		
	miR-210	mNPCs	MCAO mice	tail vein	↓infarct volume	↓Nox2	146
	miR-126				↓neurological deficits	↑BDNF	
					↓neural apoptosis	↑p-TrkB/TrkB	
					↓ROS production		
					↑spine density of dendrites		
**Surface-modification**	RGD-4C	hNPCs	MCAO mice	tail vein	↓inflammatory response	↓MAPK	33
**Other**	Hydrogel	hADMSCs	5×FAD mice	intranasally	↓memory deficits	NA	147
					↓neuronal damage		
					↑neurogenesis		
	HAD hydrogel	mNSCs	MCAO mice	ventricle	↑neurological functions	NA	148
					↑infarct volume and angiogenesis		
					↑cerebral angiogenesis and anti-inflammation		
	RVG	mBMSCs	3xTg-AD mice	intranasally	↓cognitive deficits	↓BACE1	149
	BACE1 siRNA				↓ Aβ plaques	↓caspase-3	
	caspase-3 siRNA				↓apoptosis of neurons		
					↓reactive astrocytes		
					↑AD pathologies		
	curcumin	mBMSCs	MPTP mice	intranasally	↑movement and coordination ability	NA	150
	miR-133b				↓ɑ-synuclein aggregates		
	SPIONs				↑neuron function recovery		
	RVG29				↓neuroinflammation		
	P						

h, human; r, rat; m, mouse; BMSCs, bone marrow-derived mesenchymal stem cells; MSCs, mesenchymal stem cells; UCMSCs, Umbilical cord mesenchymal stem cells; NSCs, neural stem cells; ADMSCs, adipose-derived stem cells; NPCs, neural progenitor cells; iNSCs, induced pluripotent stem cell-derived neural stem cells; 5XFAD mice, 5 familial Alzheimer's disease mutations; MCAO, middle cerebral artery occlusion; UCMSCs, umbilical cord mesenchymal stem cells; 6-OHDA, 6-hydroxydopamine; TLR4, toll-like receptor-4; PTEN, phosphatase and tensin homolog deleted on chromosome ten; Akt, protein kinase B; BHD, buyang huanwu decoction; SHP2, tyrosine phosphatase-2; Aβ, amyloid β; EGR1, growth-response-1; CDK5, cell division protein kinase 5; FTO, m6A demethylase fat mass and obesity-related protein; TH, tyrosine hydroxylase; ɑ-syn, ɑ-synuclein; mTOR, mammalian target of rapamycin; BDNF, brain-derived neurotrophic factor; Nox2, NADPH oxidase 2; TrkB, BDNF receptor tyrosine kinase receptor B; ROS, reactive oxygen species; Sirt1, silent mating type information regulation 2 homolog 1; USP22, ubiquitin specific peptidase 22; RGD, arginine-glycine-aspartic acid; MAPK, mitogen-activated protein kinase; HAD, adhesive hyaluronic acid; RVG, rabies virus glycoprotein; BACE1, β-site amyloid precursor protein cleaving enzyme 1; siRNA, small interfering RNAs; SPIONs, superparamagnetic iron oxide nanoparticles; P, penetratin; NA, not available.

## Abbreviations

Aβ: amyloid β
ɑ-syn: ɑ-synuclein
AD: Alzheimer's disease
Akt: protein kinase B
BACE1: β-site amyloid precursor protein cleaving enzyme 1
BBB: blood-brain barrier
BDNF: brain-derived neurotrophic factor
CNS: central nervous system
EB: embryoid body
EVs: extracellular vesicles
FTO: targeting obesity-associated protein
GDNF: glial cell-derived neurotrophic factor
GMP: good manufacturing practices
HBMECs: human brain microvascular endothelial cells
HUVECs: human umbilical vein endothelial cells
IEP: insulin-degrading enzyme
IL: interleukin
iMSCs: induced mesenchymal stem cells
iNSCs: induced pluripotent stem cell-derived neural stem cells
iPSCs: induced pluripotent stem cells
IS: ischemic stroke
MAPK: mitogen-activated kinase
MCAO: middle cerebral artery occlusion
MMP: matrix metalloproteinase
mTOR: mammalian target of rapamycin
NEP: neprilysin
NF-κB: nuclear factor-kappa B
NPCs: neural progenitor cells
NTFs: neurotrophic factors
OGD: oxygen and glucose deprivation
OPCs: oligodendrocyte progenitor cells
PD: Parkinson's disease
PTEN: phosphatase and tensin homolog deleted on chromosome ten
RGD: arginine-glycine-aspartic acid
ROS: reactive oxygen species
RVG: rabies virus glycoprotein
SCs: stem cells
siRNA: small interfering RNAs
Sirt: silent mating type information regulation 2 homolog
SHP2: tyrosine phosphatase-2
VEGF: vascular endothelial growth factor
VEGFR: vascular endothelial growth factor receptor

## References

[1] Jin T, Gu J, Li Z, Xu Z, Gui Y (2021). Recent Advances on Extracellular Vesicles in Central Nervous System Diseases. Clin Interv Aging.

[2] Stam CJ (2014). Modern network science of neurological disorders. Nat Rev Neurosci.

[3] Courtine G, Sofroniew MV (2019). Spinal cord repair: advances in biology and technology. Nat Med.

[4] Bacakova L, Zarubova J, Travnickova M, Musilkova J, Pajorova J, Slepicka P (2018). Stem cells: their source, potency and use in regenerative therapies with focus on adipose-derived stem cells - a review. Biotechnol Adv.

[5] Lv ZY, Li Y, Liu J (2021). Progress in clinical trials of stem cell therapy for cerebral palsy. Neural Regen Res.

[6] Sissung TM, Figg WD (2020). Stem cell clinics: risk of proliferation. Lancet Oncol.

[7] van Niel G, D'Angelo G, Raposo G (2018). Shedding light on the cell biology of extracellular vesicles. Nat Rev Mol Cell Biol.

[8] Xia X, Wang Y, Qin Y, Zhao S, Zheng JC (2022). Exosome: A novel neurotransmission modulator or non-canonical neurotransmitter?. Ageing Res Rev.

[9] Kalluri R, LeBleu VS (2020). The biology, function, and biomedical applications of exosomes. Science.

[10] Moghadasi S, Elveny M, Rahman HS, Suksatan W, Jalil AT, Abdelbasset WK (2021). A paradigm shift in cell-free approach: the emerging role of MSCs-derived exosomes in regenerative medicine. J Transl Med.

[11] Moura RP, Martins C, Pinto S, Sousa F, Sarmento B (2019). Blood-brain barrier receptors and transporters: an insight on their function and how to exploit them through nanotechnology. Expert Opin Drug Deliv.

[12] Doeppner TR, Herz J, Görgens A, Schlechter J, Ludwig AK, Radtke S (2015). Extracellular Vesicles Improve Post-Stroke Neuroregeneration and Prevent Postischemic Immunosuppression. Stem Cells Transl Med.

[13] Tieu A, Lalu MM, Slobodian M, Gnyra C, Fergusson DA, Montroy J (2020). An Analysis of Mesenchymal Stem Cell-Derived Extracellular Vesicles for Preclinical Use. ACS Nano.

[14] Li QC, Li C, Zhang W, Pi W, Han N (2022). Potential Effects of Exosomes and their MicroRNA Carrier on Osteoporosis. Curr Pharm Des.

[15] Saffari S, Saffari TM, Ulrich DJO, Hovius SER, Shin AY (2021). The interaction of stem cells and vascularity in peripheral nerve regeneration. Neural Regen Res.

[16] Mirzamohammadi S, Aali E, Najafi R, Kamarul T, Mehrabani M, Aminzadeh A, Sharifi AM (2015). Effect of 17β-estradiol on mediators involved in mesenchymal stromal cell trafficking in cell therapy of diabetes. Cytotherapy.

[17] Biehl JK, Russell B (2009). Introduction to stem cell therapy. J Cardiovasc Nurs.

[18] Luzzani CD, Miriuka SG (2017). Pluripotent Stem Cells as a Robust Source of Mesenchymal Stem Cells. Stem Cell Rev Rep.

[19] Alessandrini M, Preynat-Seauve O, De Bruin K, Pepper MS (2019). Stem cell therapy for neurological disorders. S Afr Med J.

[20] Yasuhara T, Kawauchi S, Kin K, Morimoto J, Kameda M, Sasaki T (2020). Cell therapy for central nervous system disorders: Current obstacles to progress. CNS Neurosci Ther.

[21] Gratpain V, Mwema A, Labrak Y, Muccioli GG, van Pesch V, des Rieux A (2021). Extracellular vesicles for the treatment of central nervous system diseases. Adv Drug Deliv Rev.

[22] Kourembanas S (2015). Exosomes: vehicles of intercellular signaling, biomarkers, and vectors of cell therapy. Annu Rev Physiol.

[23] Sharma P, Schiapparelli L, Cline HT (2013). Exosomes function in cell-cell communication during brain circuit development. Curr Opin Neurobiol.

[24] Ding L, Yang X, Gao Z, Effah CY, Zhang X, Wu Y, Qu L (2021). A Holistic Review of the State-of-the-Art Microfluidics for Exosome Separation: An Overview of the Current Status, Existing Obstacles, and Future Outlook. Small.

[25] Dragovic RA, Gardiner C, Brooks AS, Tannetta DS, Ferguson DJ, Hole P (2011). Sizing and phenotyping of cellular vesicles using Nanoparticle Tracking Analysis. Nanomedicine.

[26] Alenquer M, Amorim MJ (2015). Exosome Biogenesis, Regulation, and Function in Viral Infection. Viruses.

[27] Qiu G, Zheng G, Ge M, Wang J, Huang R, Shu Q, Xu J (2019). Functional proteins of mesenchymal stem cell-derived extracellular vesicles. Stem Cell Res Ther.

[28] Harding C, Heuser J, Stahl P (1983). Receptor-mediated endocytosis of transferrin and recycling of the transferrin receptor in rat reticulocytes. J Cell Biol.

[29] Ong SG, Wu JC (2015). Exosomes as potential alternatives to stem cell therapy in mediating cardiac regeneration. Circ Res.

[30] Askenase PW (2021). Ancient Evolutionary Origin and Properties of Universally Produced Natural Exosomes Contribute to Their Therapeutic Superiority Compared to Artificial Nanoparticles. Int J Mol Sci.

[31] Xia X, Wang Y, Huang Y, Zhang H, Lu H, Zheng JC (2019). Exosomal miRNAs in central nervous system diseases: biomarkers, pathological mediators, protective factors and therapeutic agents. Prog Neurobiol.

[32] Kooijmans SAA, Schiffelers RM, Zarovni N, Vago R (2016). Modulation of tissue tropism and biological activity of exosomes and other extracellular vesicles: New nanotools for cancer treatment. Pharmacol Res.

[33] Tian T, Cao L, He C, Ye Q, Liang R, You W (2021). Targeted delivery of neural progenitor cell-derived extracellular vesicles for anti-inflammation after cerebral ischemia. Theranostics.

[34] Almeida M, García-Montero AC, Orfao A (2014). Cell purification: a new challenge for biobanks. Pathobiology.

[35] Cuthbert RJ, Giannoudis PV, Wang XN, Nicholson L, Pawson D, Lubenko A (2015). Examining the feasibility of clinical grade CD271+ enrichment of mesenchymal stromal cells for bone regeneration. PLoS One.

[36] Berger C, Blau CA, Clackson T, Riddell SR, Heimfeld S (2003). CD28 costimulation and immunoaffinity-based selection efficiently generate primary gene-modified T cells for adoptive immunotherapy. Blood.

[37] Villa-Diaz LG, Brown SE, Liu Y, Ross AM, Lahann J, Parent JM, Krebsbach PH (2012). Derivation of mesenchymal stem cells from human induced pluripotent stem cells cultured on synthetic substrates. Stem Cells.

[38] Chen YS, Pelekanos RA, Ellis RL, Horne R, Wolvetang EJ, Fisk NM (2012). Small molecule mesengenic induction of human induced pluripotent stem cells to generate mesenchymal stem/stromal cells. Stem Cells Transl Med.

[39] Wen Y, Jin S (2014). Production of neural stem cells from human pluripotent stem cells. J Biotechnol.

[40] Morizane A, Doi D, Kikuchi T, Nishimura K, Takahashi J (2011). Small-molecule inhibitors of bone morphogenic protein and activin/nodal signals promote highly efficient neural induction from human pluripotent stem cells. J Neurosci Res.

[41] Surmacz B, Noisa P, Risner-Janiczek JR, Hui K, Ungless M, Cui W, Li M (2012). DLK1 promotes neurogenesis of human and mouse pluripotent stem cell-derived neural progenitors via modulating Notch and BMP signalling. Stem Cell Rev Rep.

[42] Song S, Sanchez-Ramos J (2008). Preparation of neural progenitors from bone marrow and umbilical cord blood. Methods Mol Biol.

[43] Chen J, Li P, Zhang T, Xu Z, Huang X, Wang R, Du L (2021). Review on Strategies and Technologies for Exosome Isolation and Purification. Front Bioeng Biotechnol.

[44] Böing AN, van der Pol E, Grootemaat AE, Coumans FA, Sturk A, Nieuwland R (2014). Single-step isolation of extracellular vesicles by size-exclusion chromatography. J Extracell Vesicles.

[45] Melo SA, Luecke LB, Kahlert C, Fernandez AF, Gammon ST, Kaye J (2015). Glypican-1 identifies cancer exosomes and detects early pancreatic cancer. Nature.

[46] Reiner AT, Witwer KW, van Balkom BWM, de Beer J, Brodie C, Corteling RL (2017). Concise Review: Developing Best-Practice Models for the Therapeutic Use of Extracellular Vesicles. Stem Cells Transl Med.

[47] Andriolo G, Provasi E, Lo Cicero V, Brambilla A, Soncin S, Torre T (2018). Exosomes From Human Cardiac Progenitor Cells for Therapeutic Applications: Development of a GMP-Grade Manufacturing Method. Front Physiol.

[48] Weng Y, Sui Z, Shan Y, Hu Y, Chen Y, Zhang L, Zhang Y (2016). Effective isolation of exosomes with polyethylene glycol from cell culture supernatant for in-depth proteome profiling. Analyst.

[49] Tschuschke M, Kocherova I, Bryja A, Mozdziak P, Angelova Volponi A, Janowicz K (2020). Inclusion Biogenesis, Methods of Isolation and Clinical Application of Human Cellular Exosomes. J Clin Med.

[50] Massey AE, Malik S, Sikander M, Doxtater KA, Tripathi MK, Khan S (2021). Clinical Implications of Exosomes: Targeted Drug Delivery for Cancer Treatment. Int J Mol Sci.

[51] Zarovni N, Corrado A, Guazzi P, Zocco D, Lari E, Radano G (2015). Integrated isolation and quantitative analysis of exosome shuttled proteins and nucleic acids using immunocapture approaches. Methods.

[52] Shao H, Im H, Castro CM, Breakefield X, Weissleder R, Lee H (2018). New Technologies for Analysis of Extracellular Vesicles. Chem Rev.

[53] Ford T, Graham J, Rickwood D (1994). Iodixanol: a nonionic iso-osmotic centrifugation medium for the formation of self-generated gradients. Anal Biochem.

[54] Jalaludin I, Lubman DM, Kim J (2023). A guide to mass spectrometric analysis of extracellular vesicle proteins for biomarker discovery. Mass Spectrom Rev.

[55] Rider MA, Hurwitz SN, Meckes DG Jr (2016). ExtraPEG: A Polyethylene Glycol-Based Method for Enrichment of Extracellular Vesicles. Sci Rep.

[56] Li P, Kaslan M, Lee SH, Yao J, Gao Z (2017). Progress in Exosome Isolation Techniques. Theranostics.

[57] Wang H, Liu Y, Li J, Wang T, Hei Y, Li H (2021). Tail-vein injection of MSC-derived small extracellular vesicles facilitates the restoration of hippocampal neuronal morphology and function in APP / PS1 mice. Cell Death Discov.

[58] Liu S, Fan M, Xu JX, Yang LJ, Qi CC, Xia QR, Ge JF (2022). Exosomes derived from bone-marrow mesenchymal stem cells alleviate cognitive decline in AD-like mice by improving BDNF-related neuropathology. J Neuroinflammation.

[59] Lin L, Huang L, Huang S, Chen W, Huang H, Chi L (2024). MSC-Derived Extracellular Vesicles Alleviate NLRP3/GSDMD-Mediated Neuroinflammation in Mouse Model of Sporadic Alzheimer's Disease. Mol Neurobiol.

[60] Sha S, Shen X, Cao Y, Qu L (2021). Mesenchymal stem cells-derived extracellular vesicles ameliorate Alzheimer's disease in rat models via the microRNA-29c-3p/BACE1 axis and the Wnt/β-catenin pathway. Aging (Albany NY).

[61] Cone AS, Yuan X, Sun L, Duke LC, Vreones MP, Carrier AN (2021). Mesenchymal stem cell-derived extracellular vesicles ameliorate Alzheimer's disease-like phenotypes in a preclinical mouse model. Theranostics.

[62] Li B, Chen Y, Zhou Y, Feng X, Gu G, Han S (2024). Neural stem cell-derived exosomes promote mitochondrial biogenesis and restore abnormal protein distribution in a mouse model of Alzheimer's disease. Neural Regen Res.

[63] He S, Wang Q, Chen L, He YJ, Wang X, Qu S (2023). miR-100a-5p-enriched exosomes derived from mesenchymal stem cells enhance the anti-oxidant effect in a Parkinson's disease model via regulation of Nox4/ROS/Nrf2 signaling. J Transl Med.

[64] Zhang ZX, Zhou YJ, Gu P, Zhao W, Chen HX, Wu RY (2023). Exosomes derived from human umbilical cord mesenchymal stem cells alleviate Parkinson's disease and neuronal damage through inhibition of microglia. Neural Regen Res.

[65] Xue C, Li X, Ba L, Zhang M, Yang Y, Gao Y (2021). MSC-Derived Exosomes can Enhance the Angiogenesis of Human Brain MECs and Show Therapeutic Potential in a Mouse Model of Parkinson's Disease. Aging Dis.

[66] Lee EJ, Choi Y, Lee HJ, Hwang DW, Lee DS (2022). Human neural stem cell-derived extracellular vesicles protect against Parkinson's disease pathologies. J Nanobiotechnology.

[67] Zhang Y, Liu J, Su M, Wang X, Xie C (2021). Exosomal microRNA-22-3p alleviates cerebral ischemic injury by modulating KDM6B/BMP2/BMF axis. Stem Cell Res Ther.

[68] Wang QS, Xiao RJ, Peng J, Yu ZT, Fu JQ, Xia Y (2023). Bone Marrow Mesenchymal Stem Cell-Derived Exosomal KLF4 Alleviated Ischemic Stroke Through Inhibiting N6-Methyladenosine Modification Level of Drp1 by Targeting lncRNA-ZFAS1. Mol Neurobiol.

[69] Zheng X, Zhang L, Kuang Y, Venkataramani V, Jin F, Hein K (2021). Extracellular Vesicles Derived from Neural Progenitor Cells-a Preclinical Evaluation for Stroke Treatment in Mice. Transl Stroke Res.

[70] Hu H, Hu X, Li L, Fang Y, Yang Y, Gu J (2022). Exosomes Derived from Bone Marrow Mesenchymal Stem Cells Promote Angiogenesis in Ischemic Stroke Mice via Upregulation of MiR-21-5p. Biomolecules.

[71] Li Y, Chen J, Quan X, Chen Y, Han Y, Chen J (2024). Extracellular Vesicles Maintain Blood-Brain Barrier Integrity by the Suppression of Caveolin-1/CD147/VEGFR2/MMP Pathway After Ischemic Stroke. Int J Nanomedicine.

[72] Dumbrava DA, Surugiu R, Börger V, Ruscu M, Tertel T, Giebel B (2022). Mesenchymal stromal cell-derived small extracellular vesicles promote neurological recovery and brain remodeling after distal middle cerebral artery occlusion in aged rats. Geroscience.

[73] Li Y, Liu B, Zhao T, Quan X, Han Y, Cheng Y (2023). Comparative study of extracellular vesicles derived from mesenchymal stem cells and brain endothelial cells attenuating blood-brain barrier permeability via regulating Caveolin-1-dependent ZO-1 and Claudin-5 endocytosis in acute ischemic stroke. J Nanobiotechnology.

[74] Rohden F, Teixeira LV, Bernardi LP, Ferreira PCL, Colombo M, Teixeira GR (2021). Functional Recovery Caused by Human Adipose Tissue Mesenchymal Stem Cell-Derived Extracellular Vesicles Administered 24 h after Stroke in Rats. Int J Mol Sci.

[75] Qiu L, Cai Y, Geng Y, Yao X, Wang L, Cao H (2022). Mesenchymal stem cell-derived extracellular vesicles attenuate tPA-induced blood-brain barrier disruption in murine ischemic stroke models. Acta Biomater.

[76] Zhang L, Graf I, Kuang Y, Zheng X, Haupt M, Majid A (2021). Neural Progenitor Cell-Derived Extracellular Vesicles Enhance Blood-Brain Barrier Integrity by NF-κB (Nuclear Factor-κB)-Dependent Regulation of ABCB1 (ATP-Binding Cassette Transporter B1) in Stroke Mice. Arterioscler Thromb Vasc Biol.

[77] Pathipati P, Lecuyer M, Faustino J, Strivelli J, Phinney DG, Vexler ZS (2021). Mesenchymal Stem Cell (MSC)-Derived Extracellular Vesicles Protect from Neonatal Stroke by Interacting with Microglial Cells. Neurotherapeutics.

[78] Liu C, Yang TH, Li HD, Li GZ, Liang J, Wang P (2023). Exosomes from bone marrow mesenchymal stem cells are a potential treatment for ischemic stroke. Neural Regen Res.

[79] Wang C, Börger V, Mohamud Yusuf A, Tertel T, Stambouli O, Murke F (2022). Postischemic Neuroprotection Associated With Anti-Inflammatory Effects by Mesenchymal Stromal Cell-Derived Small Extracellular Vesicles in Aged Mice. Stroke.

[80] Liu X, Zhang M, Liu H, Zhu R, He H, Zhou Y (2021). Bone marrow mesenchymal stem cell-derived exosomes attenuate cerebral ischemia-reperfusion injury-induced neuroinflammation and pyroptosis by modulating microglia M1/M2 phenotypes. Exp Neurol.

[81] Zhang Z, Zou X, Zhang R, Xie Y, Feng Z, Li F (2021). Human umbilical cord mesenchymal stem cell-derived exosomal miR-146a-5p reduces microglial-mediated neuroinflammation via suppression of the IRAK1/TRAF6 signaling pathway after ischemic stroke. Aging (Albany NY).

[82] Zhang R, Mao W, Niu L, Bao W, Wang Y, Wang Y (2023). NSC-derived exosomes enhance therapeutic effects of NSC transplantation on cerebral ischemia in mice. Elife.

[83] Peng J, He J, Lin L, Li Y, Xia Y (2023). Neural Stem Cell Extracellular Vesicles Carrying YBX1 Inhibited Neuronal Pyroptosis Through Increasing m6A-modified GPR30 Stability and Expression in Ischemic Stroke. Transl Stroke Res.

[84] Go V, Sarikaya D, Zhou Y, Bowley BGE, Pessina MA, Rosene DL (2021). Extracellular vesicles derived from bone marrow mesenchymal stem cells enhance myelin maintenance after cortical injury in aged rhesus monkeys. Exp Neurol.

[85] Hou H, Wang Y, Yang L, Wang Y (2023). Exosomal miR-128-3p reversed fibrinogen-mediated inhibition of oligodendrocyte progenitor cell differentiation and remyelination after cerebral ischemia. CNS Neurosci Ther.

[86] Bankston AN, Mandler MD, Feng Y (2013). Oligodendroglia and neurotrophic factors in neurodegeneration. Neurosci Bull.

[87] Kim DH, Lee D, Chang EH, Kim JH, Hwang JW, Kim JY (2015). GDF-15 secreted from human umbilical cord blood mesenchymal stem cells delivered through the cerebrospinal fluid promotes hippocampal neurogenesis and synaptic activity in an Alzheimer's disease model. Stem Cells Dev.

[88] Markus A, Patel TD, Snider WD (2002). Neurotrophic factors and axonal growth. Curr Opin Neurobiol.

[89] Bucan V, Vaslaitis D, Peck CT, Strauß S, Vogt PM, Radtke C (2019). Effect of Exosomes from Rat Adipose-Derived Mesenchymal Stem Cells on Neurite Outgrowth and Sciatic Nerve Regeneration After Crush Injury. Mol Neurobiol.

[90] Ahn SY, Sung DK, Kim YE, Sung S, Chang YS, Park WS (2021). Brain-derived neurotropic factor mediates neuroprotection of mesenchymal stem cell-derived extracellular vesicles against severe intraventricular hemorrhage in newborn rats. Stem Cells Transl Med.

[91] Haraszti RA, Didiot MC, Sapp E, Leszyk J, Shaffer SA, Rockwell HE (2016). High-resolution proteomic and lipidomic analysis of exosomes and microvesicles from different cell sources. J Extracell Vesicles.

[92] Martino G, Pluchino S (2006). The therapeutic potential of neural stem cells. Nat Rev Neurosci.

[93] Arai K, Jin G, Navaratna D, Lo EH (2009). Brain angiogenesis in developmental and pathological processes: neurovascular injury and angiogenic recovery after stroke. Febs j.

[94] Terstappen GC, Meyer AH, Bell RD, Zhang W (2021). Strategies for delivering therapeutics across the blood-brain barrier. Nat Rev Drug Discov.

[95] Chen WW, Zhang X, Huang WJ (2016). Role of neuroinflammation in neurodegenerative diseases (Review). Mol Med Rep.

[96] Vande Walle L, Lamkanfi M (2024). Drugging the NLRP3 inflammasome: from signalling mechanisms to therapeutic targets. Nat Rev Drug Discov.

[97] Yuyama K, Sun H, Usuki S, Sakai S, Hanamatsu H, Mioka T (2015). A potential function for neuronal exosomes: sequestering intracerebral amyloid-β peptide. FEBS Lett.

[98] Webb RL, Kaiser EE, Scoville SL, Thompson TA, Fatima S, Pandya C (2018). Human Neural Stem Cell Extracellular Vesicles Improve Tissue and Functional Recovery in the Murine Thromboembolic Stroke Model. Transl Stroke Res.

[99] Gao G, Li C, Zhu J, Sheng S, Liang Z, Fu S (2022). Induced neural stem/progenitor cell-derived extracellular vesicles promote recovery post-stroke. Clin Transl Med.

[100] Kim HY, Kwon S, Um W, Shin S, Kim CH, Park JH, Kim BS (2022). Functional Extracellular Vesicles for Regenerative Medicine. Small.

[101] Cao J, Wang B, Tang T, Lv L, Ding Z, Li Z (2020). Three-dimensional culture of MSCs produces exosomes with improved yield and enhanced therapeutic efficacy for cisplatin-induced acute kidney injury. Stem Cell Res Ther.

[102] Haraszti RA, Miller R, Stoppato M, Sere YY, Coles A, Didiot MC (2018). Exosomes Produced from 3D Cultures of MSCs by Tangential Flow Filtration Show Higher Yield and Improved Activity. Mol Ther.

[103] Zhao M, Gao Y, Wang F, Cheng X, Zhao T, Zhao Y (2020). Neural progenitor cells-secreted exosomal miR-210 induced by hypoxia influences cell viability. Neuroreport.

[104] Zhu J, Lu K, Zhang N, Zhao Y, Ma Q, Shen J (2018). Myocardial reparative functions of exosomes from mesenchymal stem cells are enhanced by hypoxia treatment of the cells via transferring microRNA-210 in an nSMase2-dependent way. Artif Cells Nanomed Biotechnol.

[105] Losurdo M, Pedrazzoli M, D'Agostino C, Elia CA, Massenzio F, Lonati E (2020). Intranasal delivery of mesenchymal stem cell-derived extracellular vesicles exerts immunomodulatory and neuroprotective effects in a 3xTg model of Alzheimer's disease. Stem Cells Transl Med.

[106] Zhang G, Zhu Z, Wang H, Yu Y, Chen W, Waqas A (2020). Exosomes derived from human neural stem cells stimulated by interferon gamma improve therapeutic ability in ischemic stroke model. J Adv Res.

[107] Ti D, Hao H, Tong C, Liu J, Dong L, Zheng J (2015). LPS-preconditioned mesenchymal stromal cells modify macrophage polarization for resolution of chronic inflammation via exosome-shuttled let-7b. J Transl Med.

[108] Fukuta T, Nishikawa A, Kogure K (2020). Low level electricity increases the secretion of extracellular vesicles from cultured cells. Biochem Biophys Rep.

[109] Zhang Y, Chopp M, Zhang ZG, Katakowski M, Xin H, Qu C (2017). Systemic administration of cell-free exosomes generated by human bone marrow derived mesenchymal stem cells cultured under 2D and 3D conditions improves functional recovery in rats after traumatic brain injury. Neurochem Int.

[110] Batrakova EV, Kim MS (2015). Using exosomes, naturally-equipped nanocarriers, for drug delivery. J Control Release.

[111] Zhuang X, Xiang X, Grizzle W, Sun D, Zhang S, Axtell RC (2011). Treatment of brain inflammatory diseases by delivering exosome encapsulated anti-inflammatory drugs from the nasal region to the brain. Mol Ther.

[112] Li BQ, Fang TL, Li Y, Xue TY, Zhang ZR, Li LY (2022). Engineered T cell extracellular vesicles displaying PD-1 boost anti-tumor immunity. Nano Today.

[113] Kalani A, Kamat PK, Chaturvedi P, Tyagi SC, Tyagi N (2014). Curcumin-primed exosomes mitigate endothelial cell dysfunction during hyperhomocysteinemia. Life Sci.

[114] Kim MS, Haney MJ, Zhao Y, Yuan D, Deygen I, Klyachko NL (2018). Engineering macrophage-derived exosomes for targeted paclitaxel delivery to pulmonary metastases: in vitro and in vivo evaluations. Nanomedicine.

[115] Kim HY, Kumar H, Jo MJ, Kim J, Yoon JK, Lee JR (2018). Therapeutic Efficacy-Potentiated and Diseased Organ-Targeting Nanovesicles Derived from Mesenchymal Stem Cells for Spinal Cord Injury Treatment. Nano Lett.

[116] Haney MJ, Klyachko NL, Zhao Y, Gupta R, Plotnikova EG, He Z (2015). Exosomes as drug delivery vehicles for Parkinson's disease therapy. J Control Release.

[117] Kumar P, Nagarajan A, Uchil PD (2019). Electroporation. Cold Spring Harb Protoc. 2019.

[118] Johnsen KB, Gudbergsson JM, Skov MN, Christiansen G, Gurevich L, Moos T, Duroux M (2016). Evaluation of electroporation-induced adverse effects on adipose-derived stem cell exosomes. Cytotechnology.

[119] Podolak I, Galanty A, Sobolewska D (2010). Saponins as cytotoxic agents: a review. Phytochem Rev.

[120] Bodart-Santos V, de Carvalho LRP, de Godoy MA, Batista AF, Saraiva LM, Lima LG (2019). Extracellular vesicles derived from human Wharton's jelly mesenchymal stem cells protect hippocampal neurons from oxidative stress and synapse damage induced by amyloid-β oligomers. Stem Cell Res Ther.

[121] Tian T, Zhang HX, He CP, Fan S, Zhu YL, Qi C (2018). Surface functionalized exosomes as targeted drug delivery vehicles for cerebral ischemia therapy. Biomaterials.

[122] Mentkowski KI, Snitzer JD, Rusnak S, Lang JK (2018). Therapeutic Potential of Engineered Extracellular Vesicles. Aaps j.

[123] Horrevorts SK, Stolk DA, van de Ven R, Hulst M, van Het Hof B, Duinkerken S (2019). Glycan-Modified Apoptotic Melanoma-Derived Extracellular Vesicles as Antigen Source for Anti-Tumor Vaccination. Cancers (Basel).

[124] Xu K, Jin Y, Li Y, Huang Y, Zhao R (2022). Recent Progress of Exosome Isolation and Peptide Recognition-Guided Strategies for Exosome Research. Front Chem.

[125] Feng Z, Xu B (2016). Inspiration from the mirror: D-amino acid containing peptides in biomedical approaches. Biomol Concepts.

[126] Hung ME, Leonard JN (2015). Stabilization of exosome-targeting peptides via engineered glycosylation. J Biol Chem.

[127] Hood JL (2016). Post isolation modification of exosomes for nanomedicine applications. Nanomedicine (Lond).

[128] Wang M, Altinoglu S, Takeda YS, Xu Q (2015). Integrating Protein Engineering and Bioorthogonal Click Conjugation for Extracellular Vesicle Modulation and Intracellular Delivery. PLoS One.

[129] Smyth T, Petrova K, Payton NM, Persaud I, Redzic JS, Graner MW (2014). Surface functionalization of exosomes using click chemistry. Bioconjug Chem.

[130] Ramasubramanian L, Kumar P, Wang A (2019). Engineering Extracellular Vesicles as Nanotherapeutics for Regenerative Medicine. Biomolecules.

[131] Markoutsa E, Mayilsamy K, Gulick D, Mohapatra SS, Mohapatra S (2022). Extracellular vesicles derived from inflammatory-educated stem cells reverse brain inflammation-implication of miRNAs. Mol Ther.

[132] Wu Q, Wu JH, Ye ZY, She W, Peng WJ, Zhang HX (2024). Exosomes from Hypoxia-treated Mesenchymal Stem Cells: Promoting Neuroprotection in Ischemic Stroke Through miR-214-3p/PTEN Mechanism. Mol Neurobiol.

[133] Gregorius J, Wang C, Stambouli O, Hussner T, Qi Y, Tertel T (2021). Small extracellular vesicles obtained from hypoxic mesenchymal stromal cells have unique characteristics that promote cerebral angiogenesis, brain remodeling and neurological recovery after focal cerebral ischemia in mice. Basic Res Cardiol.

[134] Ye YC, Chang ZH, Wang P, Wang YW, Liang J, Chen C (2022). Infarct-preconditioning exosomes of umbilical cord mesenchymal stem cells promoted vascular remodeling and neurological recovery after stroke in rats. Stem Cell Res Ther.

[135] Haupt M, Zheng X, Kuang Y, Lieschke S, Janssen L, Bosche B (2021). Lithium modulates miR-1906 levels of mesenchymal stem cell-derived extracellular vesicles contributing to poststroke neuroprotection by toll-like receptor 4 regulation. Stem Cells Transl Med.

[136] Long J, Gu C, Zhang Q, Liu J, Huang J, Li Y (2023). Extracellular vesicles from medicated plasma of Buyang Huanwu decoction-preconditioned neural stem cells accelerate neurological recovery following ischemic stroke. Front Cell Dev Biol.

[137] Xu F, Wu Y, Yang Q, Cheng Y, Xu J, Zhang Y (2022). Engineered Extracellular Vesicles with SHP2 High Expression Promote Mitophagy for Alzheimer's Disease Treatment. Adv Mater.

[138] Zhu Y, Huang R, Wang D, Yu L, Liu Y, Huang R (2023). EVs-mediated delivery of CB2 receptor agonist for Alzheimer's disease therapy. Asian J Pharm Sci.

[139] Geng Y, Long X, Zhang Y, Wang Y, You G, Guo W (2023). FTO-targeted siRNA delivery by MSC-derived exosomes synergistically alleviates dopaminergic neuronal death in Parkinson's disease via m6A-dependent regulation of ATM mRNA. J Transl Med.

[140] Zhou X, Deng X, Liu M, He M, Long W, Xu Z (2023). Intranasal delivery of BDNF-loaded small extracellular vesicles for cerebral ischemia therapy. J Control Release.

[141] Zhu ZH, Jia F, Ahmed W, Zhang GL, Wang H, Lin CQ (2023). Neural stem cell-derived exosome as a nano-sized carrier for BDNF delivery to a rat model of ischemic stroke. Neural Regen Res.

[142] Xie X, Cao Y, Dai L, Zhou D (2023). Bone marrow mesenchymal stem cell-derived exosomal lncRNA KLF3-AS1 stabilizes Sirt1 protein to improve cerebral ischemia/reperfusion injury via miR-206/USP22 axis. Mol Med.

[143] Ma J, Shi X, Li M, Chen S, Gu Q, Zheng J (2022). MicroRNA-181a-2-3p shuttled by mesenchymal stem cell-secreted extracellular vesicles inhibits oxidative stress in Parkinson's disease by inhibiting EGR1 and NOX4. Cell Death Discov.

[144] Li Q, Wang Z, Xing H, Wang Y, Guo Y (2021). Exosomes derived from miR-188-3p-modified adipose-derived mesenchymal stem cells protect Parkinson's disease. Mol Ther Nucleic Acids.

[145] Xin H, Liu Z, Buller B, Li Y, Golembieski W, Gan X (2021). MiR-17-92 enriched exosomes derived from multipotent mesenchymal stromal cells enhance axon-myelin remodeling and motor electrophysiological recovery after stroke. J Cereb Blood Flow Metab.

[146] Xu X, Zhang H, Li J, Chen Y, Zhong W, Chen Y, Ma X (2023). Combination of EPC-EXs and NPC-EXs with miR-126 and miR-210 overexpression produces better therapeutic effects on ischemic stroke by protecting neurons through the Nox2/ROS and BDNF/TrkB pathways. Exp Neurol.

[147] Huang M, Zheng M, Song Q, Ma X, Zhang Q, Chen H (2024). Comparative Proteomics Inspired Self-Stimulated Release Hydrogel Reinforces the Therapeutic Effects of MSC-EVs on Alzheimer's Disease. Adv Mater.

[148] Gu C, Li Y, Liu J, Liu S, Long J, Zhang Q (2023). Neural stem cell-derived exosomes-loaded adhesive hydrogel controlled-release promotes cerebral angiogenesis and neurological function in ischemic stroke. Exp Neurol.

[149] Li J, Peng H, Zhang W, Li M, Wang N, Peng C (2023). Enhanced Nose-to-Brain Delivery of Combined Small Interfering RNAs Using Lesion-Recognizing Nanoparticles for the Synergistic Therapy of Alzheimer's Disease. ACS Appl Mater Interfaces.

[150] Peng H, Li Y, Ji W, Zhao R, Lu Z, Shen J (2022). Intranasal Administration of Self-Oriented Nanocarriers Based on Therapeutic Exosomes for Synergistic Treatment of Parkinson's Disease. ACS Nano.

[151] Sharma P, Srivastava P, Seth A, Tripathi PN, Banerjee AG, Shrivastava SK (2019). Comprehensive review of mechanisms of pathogenesis involved in Alzheimer's disease and potential therapeutic strategies. Prog Neurobiol.

[152] Murphy MP, LeVine H 3rd (2010). Alzheimer's disease and the amyloid-beta peptide. J Alzheimers Dis.

[153] Stakos DA, Stamatelopoulos K, Bampatsias D, Sachse M, Zormpas E, Vlachogiannis NI (2020). The Alzheimer's Disease Amyloid-Beta Hypothesis in Cardiovascular Aging and Disease: JACC Focus Seminar. J Am Coll Cardiol.

[154] Arranz AM, De Strooper B (2019). The role of astroglia in Alzheimer's disease: pathophysiology and clinical implications. Lancet Neurol.

[155] Zhai L, Shen H, Sheng Y, Guan Q (2021). ADMSC Exo-MicroRNA-22 improve neurological function and neuroinflammation in mice with Alzheimer's disease. J Cell Mol Med.

[156] Armstrong MJ, Okun MS (2020). Diagnosis and Treatment of Parkinson Disease: A Review. Jama.

[157] Michel PP, Hirsch EC, Hunot S (2016). Understanding Dopaminergic Cell Death Pathways in Parkinson Disease. Neuron.

[158] Eriksen JL, Wszolek Z, Petrucelli L (2005). Molecular pathogenesis of Parkinson disease. Arch Neurol.

[159] Gelb DJ, Oliver E, Gilman S (1999). Diagnostic criteria for Parkinson disease. Arch Neurol.

[160] Raza C, Anjum R, Shakeel NUA (2019). Parkinson's disease: Mechanisms, translational models and management strategies. Life Sci.

[161] Parmar M, Grealish S, Henchcliffe C (2020). The future of stem cell therapies for Parkinson disease. Nat Rev Neurosci.

[162] Barthels D, Das H (2020). Current advances in ischemic stroke research and therapies. Biochim Biophys Acta Mol Basis Dis.

[163] Liu R, Pan M-X, Tang J-C, Zhang Y, Liao H-B, Zhuang Y (2017). Role of neuroinflammation in ischemic stroke. Neuroimmunol Neuroinflamm.

[164] Coull AJ, Lovett JK, Rothwell PM (2004). Population based study of early risk of stroke after transient ischaemic attack or minor stroke: implications for public education and organisation of services. Bmj.

[165] Jivan K, Ranchod K, Modi G (2013). Management of ischaemic stroke in the acute setting: review of the current status. Cardiovasc J Afr.

[166] Jayaraj RL, Azimullah S, Beiram R, Jalal FY, Rosenberg GA (2019). Neuroinflammation: friend and foe for ischemic stroke. J Neuroinflammation.

[167] Wu XQ, Guo WH, Wang L, Xu YC, Wang ZH, Yang Y (2022). An Injectable Asymmetric-Adhesive Hydrogel as a GATA6 Cavity Macrophage Trap to Prevent the Formation of Postoperative Adhesions after Minimally Invasive Surgery. Advanced Functional Materials.

[168] Le Guellec S, Ehrmann S, Vecellio L (2021). In vitro - in vivo correlation of intranasal drug deposition. Adv Drug Deliv Rev.

[169] Scherer T, Sakamoto K, Buettner C (2021). Brain insulin signalling in metabolic homeostasis and disease. Nat Rev Endocrinol.

[170] Keller LA, Merkel O, Popp A (2022). Intranasal drug delivery: opportunities and toxicologic challenges during drug development. Drug Deliv Transl Res.

[171] Wang Q, Peng S, Hu Y, Wong CH, Kwan KM, Chan HYE, Zuo Z (2019). Efficient brain uptake and distribution of an expanded CAG RNA inhibitor DB213 via intranasal administration. Eur J Pharm Sci.

[172] Lapidus KA, Levitch CF, Perez AM, Brallier JW, Parides MK, Soleimani L (2014). A randomized controlled trial of intranasal ketamine in major depressive disorder. Biol Psychiatry.

[173] Betzer O, Perets N, Angel A, Motiei M, Sadan T, Yadid G (2017). In Vivo Neuroimaging of Exosomes Using Gold Nanoparticles. ACS Nano.

[174] Lochhead JJ, Thorne RG (2012). Intranasal delivery of biologics to the central nervous system. Adv Drug Deliv Rev.

[175] Zhou T, Yuan Z, Weng J, Pei D, Du X, He C, Lai P (2021). Challenges and advances in clinical applications of mesenchymal stromal cells. J Hematol Oncol.

[176] Li Y, Chen YH, Liu BY, Nie Q, Li LJ, Duan X (2023). Deciphering the Heterogeneity Landscape of Mesenchymal Stem/Stromal Cell-Derived Extracellular Vesicles for Precise Selection in Translational Medicine. Adv Healthc Mater.

[177] de Almeida Fuzeta M, Bernardes N, Oliveira FD, Costa AC, Fernandes-Platzgummer A, Farinha JP (2020). Scalable Production of Human Mesenchymal Stromal Cell-Derived Extracellular Vesicles Under Serum-/Xeno-Free Conditions in a Microcarrier-Based Bioreactor Culture System. Front Cell Dev Biol.

[178] Bari E, Perteghella S, Catenacci L, Sorlini M, Croce S, Mantelli M (2019). Freeze-dried and GMP-compliant pharmaceuticals containing exosomes for acellular mesenchymal stromal cell immunomodulant therapy. Nanomedicine (Lond).

[179] Watson DC, Yung BC, Bergamaschi C, Chowdhury B, Bear J, Stellas D (2018). Scalable, cGMP-compatible purification of extracellular vesicles carrying bioactive human heterodimeric IL-15/lactadherin complexes. J Extracell Vesicles.

[180] Son JP, Kim EH, Shin EK, Kim DH, Sung JH, Oh MJ (2023). Mesenchymal Stem Cell-Extracellular Vesicle Therapy for Stroke: Scalable Production and Imaging Biomarker Studies. Stem Cells Transl Med.

[181] Han M, Zhang Z, Liu Z, Liu Y, Zhao H, Wang B (2023). Three-dimensional-cultured MSC-derived exosome with hydrogel for cerebral ischemia repair. Biomater Adv.

[182] Gelibter S, Marostica G, Mandelli A, Siciliani S, Podini P, Finardi A, Furlan R (2022). The impact of storage on extracellular vesicles: A systematic study. J Extracell Vesicles.

